# Racial Disparities in COVID-19 Outcomes Among Black and White Patients With Cancer

**DOI:** 10.1001/jamanetworkopen.2022.4304

**Published:** 2022-03-28

**Authors:** Julie Fu, Sonya A. Reid, Benjamin French, Cassandra Hennessy, Clara Hwang, Na Tosha Gatson, Narjust Duma, Sanjay Mishra, Ryan Nguyen, Jessica E. Hawley, Sunny R. K. Singh, David D. Chism, Neeta K. Venepalli, Jeremy L. Warner, Toni K. Choueiri, Andrew L. Schmidt, Leslie A. Fecher, Jennifer E. Girard, Mehmet A. Bilen, Deepak Ravindranathan, Sharad Goyal, Trisha M. Wise-Draper, Cathleen Park, Corrie A. Painter, Sheila M. McGlown, Gilberto de Lima Lopes, Oscar K. Serrano, Dimpy P. Shah

**Affiliations:** 1 Department of Internal Medicine, Hematology-Oncology, Tufts Medical Center Cancer Center, Stoneham, Massachusetts; 2 Division of Hematology/Oncology, Department of Medicine, Vanderbilt University, Nashville, Tennessee; 3 Vanderbilt-Ingram Cancer Center at Vanderbilt University Medical Center, Nashville, Tennessee; 4 Department of Biostatistics, Vanderbilt University, Nashville, Tennessee; 5 Department of Internal Medicine, Division of Hematology-Oncology, Henry Ford Cancer Institute, Detroit, Michigan; 6 Geisinger Health System, Danville, Danville, Pennsylvania; 7 Department of Cancer Medicine, Division of Neuro-Oncology, Banner MD Anderson Cancer Center, Gilbert, Arizona; 8 Division of Medical Oncology, Department of Medicine, Dana-Farber Cancer Institute, Boston, Massachusetts; 9 Department of Hematology and Oncology, University of Illinois, Chicago; 10 Herbert Irving Comprehensive Cancer Center at Columbia University, New York, New York; 11 Now with Division of Oncology, Fred Hutchinson Cancer Research Center, University of Washington, Seattle; 12 Thompson Cancer Survival Center, Knoxville, Tennessee; 13 Division of Oncology, Department of Medicine, University of North Carolina, Chapel Hill; 14 Department of Biomedical Informatics, Vanderbilt University, Nashville, Tennessee; 15 Rogel Cancer Center, University of Michigan, Ann Arbor; 16 Department of Hematology and Medical Oncology, Winship Cancer Institute of Emory University, Atlanta, Georgia; 17 Department of Radiation Oncology, George Washington University, Washington, DC; 18 Department of Internal Medicine, Division of Hematology-Oncology, University of Cincinnati Cancer Center, Cincinnati, Ohio; 19 Department of Hematology-Oncology, University of California, Davis; 20 Count Me In, Broad Institute of MIT and Harvard, Cambridge, Massachusetts; 21 Patient advocate; 22 Sylvester Comprehensive Cancer Center at the University of Miami Miller School of Medicine, Miami, Florida; 23 Department of Surgery, Hartford HealthCare Cancer Institute, Hartford, Connecticut; 24 Population Health Sciences, Mays Cancer Center at University of Texas Health San Antonio MD Anderson, San Antonio

## Abstract

**Importance:**

Non-Hispanic Black individuals experience a higher burden of COVID-19 than the general population; hence, there is an urgent need to characterize the unique clinical course and outcomes of COVID-19 in Black patients with cancer.

**Objective:**

To investigate racial disparities in severity of COVID-19 presentation, clinical complications, and outcomes between Black patients and non-Hispanic White patients with cancer and COVID-19.

**Design, Setting, and Participants:**

This retrospective cohort study used data from the COVID-19 and Cancer Consortium registry from March 17, 2020, to November 18, 2020, to examine the clinical characteristics and outcomes of COVID-19 in Black patients with cancer. Data analysis was performed from December 2020 to February 2021.

**Exposures:**

Black and White race recorded in patient’s electronic health record.

**Main Outcomes and Measures:**

An a priori 5-level ordinal scale including hospitalization intensive care unit admission, mechanical ventilation, and all-cause death.

**Results:**

Among 3506 included patients (1768 women [50%]; median [IQR] age, 67 [58-77] years), 1068 (30%) were Black and 2438 (70%) were White. Black patients had higher rates of preexisting comorbidities compared with White patients, including obesity (480 Black patients [45%] vs 925 White patients [38%]), diabetes (411 Black patients [38%] vs 574 White patients [24%]), and kidney disease (248 Black patients [23%] vs 392 White patients [16%]). Despite the similar distribution of cancer type, cancer status, and anticancer therapy at the time of COVID-19 diagnosis, Black patients presented with worse illness and had significantly worse COVID-19 severity (unweighted odds ratio, 1.34 [95% CI, 1.15-1.58]; weighted odds ratio, 1.21 [95% CI, 1.11-1.33]).

**Conclusions and Relevance:**

These findings suggest that Black patients with cancer experience worse COVID-19 outcomes compared with White patients. Understanding and addressing racial inequities within the causal framework of structural racism is essential to reduce the disproportionate burden of diseases, such as COVID-19 and cancer, in Black patients.

## Key Points

**Question:**

Among patients with cancer and COVID-19, do non-Hispanic Black patients have more severe COVID-19 at presentation and worse COVID-19–related outcomes compared with non-Hispanic White patients, after adjusting for demographic and clinical risk factors?

**Findings:**

In this cohort study of 3506 patients, Black patients with cancer experienced significantly more severe COVID-19 outcomes compared with White patients with cancer, after adjustment for demographic and clinical risk factors.

**Meaning:**

These findings suggest that, within the framework of structural racism in the US, having cancer and COVID-19 is associated with worse outcomes among Black patients compared with White patients.

## Teaser

This cohort study investigates racial disparities in the severity of COVID-19 presentation, clinical complications, and outcomes between non-Hispanic Black and non-Hispanic White patients with cancer and COVID-19.

## Introduction

The COVID-19 pandemic has led to an estimated 33 million cases and more than 604 000 COVID-19–related deaths in the US alone (as of July 16, 2021).^1^ Racial and ethnic minority groups, particularly non-Hispanic Black individuals, bear a disproportionate burden of COVID-19, with higher rates of infection, hospitalization, and death compared with non-Hispanic White individuals.^2,3,4,5,6^ Although Black individuals represent 13% of the US population, they account for 20% of COVID-19 cases and 23% of COVID-19–related deaths.^6^ Observations of disparate racial and ethnic burden of COVID-19 have been broadly documented across geographical regions.^7,8,9,10,11^

Comorbidities, including cancer, predispose patients to an increased risk of severe COVID-19 illness and death.^12,13,14^ At present, detailed data on racial disparities with respect to baseline prognostic factors, illness course, and outcomes among patients with cancer are limited.^12^ Before the COVID-19 pandemic, it was well described that Black patients with cancer have the highest death rates compared with all other racial and ethnic groups.^15^ Factors associated with racial disparities among patients with cancer are complex and likely constitute an interplay of socioeconomic status, preexisting comorbid conditions, access to care, and other social determinants of health (SDOH). Racial and ethnic minority groups have long experienced cancer health disparities, with a disproportionately higher burden of exposure to factors known to be associated with increased cancer risk (eg, smoking, obesity, and unhealthy diet), decreased screening and cancer prevention (and, thus, delayed cancer detection), and fewer opportunities to receive advances in cancer treatment or standard of care.^6^ Environmental, genetic, biological, behavioral, clinical, social, psychological, and cultural factors may contribute as well.^16,17,18^ Hence, we hypothesized that US Black patients with cancer and COVID-19 have worse baseline clinical characteristics, severity of COVID-19 presentation, complications, and outcomes compared with White patients after adjusting for demographic and clinical risk factors.

## Methods

### Study Population

The COVID-19 and Cancer Consortium (CCC19) registry (eAppendix 1 and eTable 1 in Supplement 1) captures detailed clinical characteristics, course of illness, and outcomes of COVID-19 among patients with cancer.^13,19,20^ Our target population included any patient with cancer and confirmed diagnosis of COVID-19 cared for at one of our participating institutions (eAppendix 2 in Supplement 1). For this registry-based, retrospective cohort study, we included all reports of laboratory-confirmed SARS-CoV-2 infection submitted to the CCC19 registry between March 17, 2020, and November 18, 2020, for US residents with current or past diagnosis of cancer and Black or White race. Race and ethnicity were derived from the patient’s electronic health record (EHR), which was either self-reported or assigned by health care practitioner or triage personnel. Excluded cancers were precursor hematological malignant neoplasms (eg, monoclonal gammopathy of undetermined significance), in situ carcinoma (except bladder), and nonmelanomatous, noninvasive skin cancer^19^ (eFigure 1 in Supplement 1). This study was approved by the Vanderbilt University institutional review board and participating sites. Informed consent was waived because the data were anonymous, and the study posed minimal risk to participants, in accordance with 45 CFR §46. Reporting of results follows the Strengthening the Reporting of Observational Studies in Epidemiology (STROBE) reporting guideline.^21^

### Study Framework

Patient-reported race and ethnicity were captured following the Centers for Disease Control and Prevention Race and Ethnicity codes.^22^ The primary outcome was a 5-level ordinal scale of COVID-19 severity based on a patient’s most severe disease status: (1) none of the complications listed here, (2) hospital admission, (3) intensive care unit admission, (4), mechanical ventilation use, and (5) death from any cause.^23^ Index date for analysis was defined as diagnosis of COVID-19, and outcomes were assessed over the patients’ total follow-up period. We performed an additional analysis with 30-day all-cause mortality as a secondary outcome.

The Institute of Medicine defines disparity as a difference in treatment provided to members of different racial (or ethnic) groups that is not due to access-related factors or needs or justified by the underlying health conditions or preferences of patients.^24^ Implementing the Institute of Medicine’s definition of disparity,^24^ only those factors not considered to be associated with disparity (ie, allowable covariates) were included in the analysis to account for the direction of the associations.^25^

Allowable covariates included demographic variables (age and sex); health behaviors (smoking status); functional status (Eastern Cooperative Oncology Group performance status, obesity, diabetes, kidney disease, pulmonary, and cardiovascular comorbidities); underlying malignant neoplasms (type of malignant neoplasm [solid or hematological], cancer status at the time of COVID-19 diagnosis [remission or no evidence of disease, active responding to therapy, stable, or progressing]); disease management (timing and modality of anticancer therapy before COVID-19 diagnosis, such as cytotoxic chemotherapy, immunotherapy, targeted therapy, endocrine therapy, locoregional therapy, radiotherapy and/or surgery with or without nodal dissection, and other); and pandemic dynamics (US Census region of patient’s residence and month of COVID-19 diagnosis to accommodate spatiotemporal trends and evolution in the clinical care and management of patients with COVID-19). The CCC19 data dictionary has been published elsewhere.^26^ Unavailable or unmeasured variables included ancestry, genetics, structural racism or racialization, societal factors (eg, language, culture, and community dynamics), physical environment (eg, air pollution, overcrowding, and green space), and access to care (eg, health insurance or enrollment in clinical trials).

### Statistical Analysis

A statistical analysis plan including model specification was predetermined before the analysis and was revised upon submission by the lead authors and the CCC19 Epidemiology, Biostatistics, and Informatics Cores (eAppendix 3 and eAppendix 4 in Supplement 1). We used standard descriptive statistics to summarize baseline covariates, rates of clinical complications (eg, cardiovascular and pulmonary complications or acute kidney injury), interventions after COVID-19 diagnosis (ie, supplemental oxygen, transfusion, remdesivir, hydroxychloroquine, and corticosteroids), and individual components of the ordinal severity outcome. We calculated unweighted and weighted absolute standardized mean differences for baseline covariates to evaluate the balance between Black and White patients; an absolute standardized mean difference less than 0.1 indicated balance.

Racial disparities in COVID-19 severity were estimated from minimally and fully adjusted multivariable ordinal logistic regression models and 30-day all-cause mortality from logistic and modified Poisson regression models.^27,28^ Treatment variables were binary indicators to account for patients receiving multiple therapies (eTable 2 in Supplement 1). The assumed functional form for continuous variables (age) was based on exploratory analyses (eFigure 2 in Supplement 1). Race-stratified estimates for obesity, comorbidities, and cancer status were obtained from adjusted models with interaction terms between race and these factors, given their known association with cancer and COVID-19 health disparities.^6^

In addition, we estimated inverse probability of treatment weighted differences in COVID-19 severity between Black and White patients from an ordinal logistic regression model and 30-day mortality from both a logistic regression model (to estimate odds ratios [ORs]) and a modified Poisson regression model (to estimate relative risks).^28^ All models included race as the sole covariate, were weighted by the reciprocal of the probability of receiving the treatment (ie, race) that was actually received, and used a robust (ie, sandwich) variance estimator to account for the uncertainty due to estimation of the weights (and for the modified Poisson model, to account for misspecification of the variance structure).

We used the e-value to quantify sensitivity to unmeasured confounding.^29,30^ Additional details on evaluating proportional odds assumptions (eFigure 3 in Supplement 1), multiple imputation to impute missing and unknown data (10 iterations; missingness rates were <5%), and additional statistical methods are provided in the eAppendix 4 in Supplement 1.^31^ Analyses were performed using R statistical software version 4.0.3 (R Project for Statistical Computing), including the Hmisc, rms, MASS, and robust extension packages. Data analysis was performed from December 2020 to February 2021.

## Results

### Patient Characteristics

From 3506 patients (1768 women [50%]; median [IQR] age, 67 [58-77] years) included in the analysis (eFigure 1 in Supplement 1), 1068 (30%) were Black and 2438 (70%) were White (Table 1). The median (IQR) duration of follow-up was 42 (29-90) days for the overall cohort, 63 (27-90) days for Black patients, and 42 (30-90) days for White patients. The median (IQR) age at COVID-19 diagnosis was 65 (57-74) for Black patients and 68 (58-78) years for White patients. At the time of COVID-19 diagnosis, Black patients had higher rates of obesity (480 Black patients [45%] vs 925 White patients [38%]), diabetes (411 Black patients [38%] vs 574 White patients [24%]), and kidney disease (248 Black patients [23%] vs 392 White patients [16%]) compared with White patients. White patients had higher rates of cardiovascular disease compared with Black patients (907 White patients [37%] vs 338 Black patients [32%]).

**Table 1.** Baseline Characteristics of Non-Hispanic Black and Non-Hispanic White Patients With Cancer and COVID-19 Diagnosis

Characteristic	Participants, No. (%)			Absolute standardized mean difference	
	Total (N = 3506)	Black (n = 1068)	White (n = 2438)	Unweighted	Weighted
Age, median (IQR), y^a^	67 (58-77)	65 (57-74)	68 (58-78)	0.163	0.046
Sex					
Female	1768 (50)	556 (52)	1212 (50)	0.048	0.023
Male	1736 (50)	511 (48)	1225 (50)	0.048	0.023
Missing or unknown^b^	2 (<1)	1 (<1)	1 (<1)	NA	NA
Smoking status					
Never	1742 (50)	532 (50)	1210 (50)	0.012	0.005
Ever	1647 (47)	496 (46)	1161 (48)	0.012	0.005
Missing or unknown^b^	107 (3)	40 (4)	67 (3)	NA	NA
Obesity^c^					
No	2075 (59)	581 (54)	1494 (61)	0.139	0.004
Yes	1405 (40)	480 (45)	925 (38)	0.139	0.004
Missing or unknown^b^	26 (1)	7 (1)	19 (1)	NA	NA
Comorbidities^d^					
Cardiovascular	1245 (36)	338 (32)	907 (37)	0.117	0.007
Pulmonary	822 (23)	246 (23)	576 (24)	0.012	0.003
Kidney disease	640 (18)	248 (23)	392 (16)	0.180	0.002
Diabetes	985 (28)	411 (38)	574 (24)	0.328	0.069
Missing or unknown^b^	33 (1)	10 (1)	23 (1)	NA	NA
Charlson Comorbidity Index score, median (IQR)^e^	1 (0-2)	1 (0-3)	1 (0-2)	0.190	0.061
Type of malignant neoplasm^d^					
Solid tumor	2886 (82)	869 (81)	2017 (83)	0.036	0.012
Hematological neoplasm	745 (21)	228 (21)	517 (21)	0.003	0.024
Eastern Cooperative Oncology Group performance status					
0	1192 (34)	368 (34)	824 (34)	0.014	0.002
1	903 (26)	316 (30)	587 (24)	0.128	0.019
≥2	586 (17)	185 (17)	401 (16)	0.024	0.008
Unknown^b^	820 (23)	196 (18)	624 (26)	0.175	0.028
Missing^b^	5 (<1)	3 (<1)	2 (<1)	NA	NA
Cancer status					
Remission or no evidence of disease	1901 (54)	555 (52)	1346 (55)	0.064	0.035
Active and responding	352 (10)	123 (12)	229 (9)	0.070	0.008
Active and stable	551 (16)	160 (15)	391 (16)	0.029	0.012
Active and progressing	432 (12)	140 (13)	292 (12)	0.035	0.011
Unknown^b^	266 (8)	88 (8)	178 (7)	0.035	0.027
Missing^b^	4 (<1)	2 (<1)	2 (<1)	NA	NA
Timing of anticancer therapy					
Never treated	276 (8)	76 (7)	200 (8)	0.049	0.029
0-4 wk before COVID-19 diagnosis	1046 (30)	352 (33)	694 (28)	0.098	0.006
1-3 mo before COVID-19 diagnosis	256 (7)	72 (7)	184 (8)	0.031	0.017
>3 mo before COVID-19 diagnosis	1763 (50)	523 (49)	1240 (51)	0.038	0.017
Missing or unknown^b^	165 (5)	45 (4)	120 (5)	NA	NA
Modality of active anticancer therapy^f^^,^^g^					
None	2076 (59)	603 (56)	1473 (60)	0.080	0.016
Cytotoxic chemotherapy	504 (14)	178 (17)	326 (13)	0.094	0.012
Targeted therapy	444 (13)	153 (14)	291 (12)	0.076	0.005
Endocrine therapy	344 (10)	106 (10)	238 (10)	0.010	0.004
Immunotherapy	165 (5)	36 (3)	129 (5)	0.092	0.005
Locoregional therapy	309 (9)	81 (8)	228 (9)	0.065	0.011
Other	25 (1)	10 (1)	15 (1)	0.036	0.001
Missing or unknown^b^	128 (4)	41 (4)	87 (4)	NA	NA
Insurance					
Medicaid alone	138 (4)	70 (7)	68 (3)	0.179	0.179
Medicare alone	895 (26)	257 (24)	638 (26)	0.049	0.020
Medicare or Medicaid with or without other	78 (2)	31 (3)	47 (2)	0.064	0.082
Other government with or without other	52 (1)	24 (2)	28 (1)	0.085	0.116
Private with or without other	909 (26)	215 (20)	694 (28)	0.195	0.192
Uninsured	33 (1)	14 (1)	19 (1)	0.052	0.038
Missing or unknown^b^	1401 (40)	457 (43)	944 (39)	NA	NA
Month of COVID-19 diagnosis					
January to April 2020	1369 (39)	460 (43)	909 (37)	0.114	0.030
May to August 2020	1796 (51)	565 (53)	1231 (50)	0.044	0.011
September to November 2020	324 (9)	41 (4)	283 (12)	0.294	0.066
Missing or unknown^b^	17 (<1)	2 (<1)	15 (1)	NA	NA
Region of patient’s residence					
Northeast	1035 (30)	284 (27)	751 (31)	0.088	0.003
Midwest	1294 (37)	426 (40)	868 (36)	0.093	0.012
South	688 (20)	296 (28)	392 (16)	0.284	0.002
West	415 (12)	44 (4)	371 (15)	0.382	0.015
Not designated	74 (2)	18 (2)	56 (2)	0.044	0.013

Abbreviation: NA, not applicable.

^a^ For patients younger than 18 years, age was truncated to 18 years; for patients older than 89 years, age was truncated to 90 years. Truncation was done in concordance with the Health Insurance Portability and Accountability Act of 1996 and to reduce the risk of reidentifiability.

^b^ The missing or unknown category indicates either missingness because of nonresponse for optional survey questions or a response of unknown; an unknown category was provided for all survey questions. For the missing or unknown category, standardized mean differences are not provided because these were calculated from imputed data.

^c^ Refers to patients reported to have obesity or to have a body mass index (calculated as weight in kilograms divided by height in meters squared) greater than 30.

^d^ Percentages could sum to greater than 100% because categories are not mutually exclusive.

^e^ Modified Klabunde index is used. Klabunde is a modification of the Charlson Comorbidity Index.

^f^ Refers to within 3 months before COVID-19 diagnosis.

^g^ Five percent of patients (n = 188) were receiving radiation treatment (52 Black patients [5%] and 136 White patients [6%]).

Most malignant neoplasms were solid tumors, with breast cancer (707 patients [20%]) being the most common solid tumor overall across both racial groups. No differences between types of cancer were observed between the 2 racial groups (eTable 3 in Supplement 1). Cancer status was similar between the 2 racial groups, with 555 Black patients (52%) and 1346 White patients (55%) in remission. Proportions of patients with active and responding (123 Black patients [12%] and 229 White patients [9%]), active and stable (160 Black patients [15%] and 391 White patients [16%]), active and progressing (140 Black patients [13%] and 292 White patients [12%]), and unknown cancer status (88 Black patients [8%] and 178 White patients [7%]) were similar between the 2 racial groups (Table 1). The proportions of patients with localized (597 Black patients [56%] and 1357 White patients [56%]), disseminated (266 Black patients [25%] and 552 White patients [23%]), or missing or unknown cancer stage (205 Black patients [19%] and 529 White patients [22%]) were also similar between Black and White patients. Almost one-half of patients had not received anticancer therapy within 3 months of COVD-19 diagnosis in both racial groups (523 Black patients [49%] and 1240 White patients [51%]). Additional baseline characteristics are shown in Table 1 and in eTable 2, eTable 3, and eTable 4 in Supplement 1.

### COVID-19–Related Clinical Outcomes, Complications, and Interventions

Black patients were more likely than White patients to have moderate (437 Black patients [41%] vs 826 White patients [34%]) or severe (164 Black patients [15%] vs 262 White patients [11%]) disease at COVID-19 diagnosis. Pulmonary complications were the most common (1289 patients [37%]), with higher rates observed among Black patients (442 patients [42%]) than White patients (847 patients [35%]) (Table 2). Similarly, Black patients had higher rates of acute kidney injury (275 Black patients [27%] vs 349 White patients [15%]) and cardiovascular complications (267 Black patients [26%] vs 526 White patients [22%]) compared with White patients (Table 2 and eTable 5 in Supplement 1). Black patients were less likely than White patients to be treated with remdesivir (61 Black patients [6%] vs 242 White patients [10%]) but were more likely to be treated with hydroxychloroquine (245 Black patients [24%] vs 348 White patients [15%]), which is consistent with our prior report on a smaller subset of patients.^23^

**Table 2.** Rates of Baseline Severity, Outcomes, Clinical Complications, and Interventions Received After COVID-19 Diagnosis

Variable	All patients (N = 3506)		Black patients		White patients	
	No.^a^	No. (%)	No.^a^	No. (%)	No.^a^	No. (%)
Baseline severity^b^						
Mild (no hospitalization indicated)	3480	1775 (51)	1062	461 (43)	2402	1314 (55)
Moderate (hospitalization indicated)	3480	1236 (36)	1062	437 (41)	2402	826 (34)
Severe (ICU admission indicated)	3480	426 (12)	1062	164 (15)	2402	262 (11)
Outcomes						
All-cause mortality						
Total^c^	3485	618 (18)	1060	206 (19)	2425	412 (17)
30-d^d^	3506	507 (14)	1068	181 (17)	2438	326 (13)
Received mechanical ventilation^c^	3495	412 (12)	1065	179 (17)	2430	233 (10)
Admitted to an ICU^c^	3444	622 (18)	1057	238 (23)	2387	384 (16)
Admitted to the hospital^c^	3505	2026 (58)	1068	696 (65)	2437	1330 (55)
Clinical complications						
Any cardiovascular complication^e^	3379	793 (23)	1028	267 (26)	2351	526 (22)
Any pulmonary complication^e^	3441	1289 (37)	1051	442 (42)	2390	847 (35)
Any gastrointestinal complication^e^	3337	129 (4)	1016	52 (5)	2321	77 (3)
Acute kidney injury	3376	624 (18)	1029	275 (27)	2347	349 (15)
Multisystem organ failure	3420	216 (6)	1040	101 (10)	2380	115 (5)
Coinfection	3270	353 (11)	1004	183 (18)	2290	450 (20)
Sepsis	3425	448 (13)	1042	170 (16)	2383	278 (12)
Any bleeding	3419	122 (4)	1041	51 (5)	2378	71 (3)
Disseminated intravascular coagulation	3414	13 (<1)	1039	7 (1)	2375	6 (<1)
Interventions						
Supplemental oxygen	3440	1511 (44)	1054	501 (48)	2386	1010 (42)
Transfusion	3287	252 (8)	1001	102 (10)	2286	150 (7)
Remdesivir	3326	303 (9)	1020	61 (6)	2306	242 (10)
Hydroxychloroquine	3323	593 (18)	1020	245 (24)	2303	348 (15)
Corticosteroids	3322	518 (16)	1020	175 (17)	2302	343 (15)
Other anti-COVID-19 treatments	3324	827 (25)	1020	278 (27)	2304	549 (24)

Abbreviation: ICU, intensive care unit.

^a^ Refers to number of patients with nonmissing data.

^b^ Mild denotes no hospitalization indicated, moderate denotes hospitalization indicated whether or not it occurred, and severe denotes ICU admission indicated, whether or not it occurred.

^c^ Included in primary ordinal COVID-19 severity outcome.

^d^ Refers to secondary outcome.

^e^ A full description of these complications is provided in eTable 4 in Supplement 1 and do not include “other.”

Higher rates of hospitalization, intensive care unit admission, and mechanical ventilation were observed among Black patients compared with White patients (Table 2). In addition, a higher rate of all-cause mortality was observed among Black patients (206 patients [19%]) compared with White patients (412 patients [17%]). Of the 618 patients who died during follow-up, 507 (82%) died within 30 days of COVID-19 diagnosis, with a 30-day mortality rate of 17% (181 patients) among Black patients and 13% (326 patients) among White patients.

### Multivariable Regression Analysis for Disease Severity and Mortality

Multivariable models revealed significantly higher COVID-19 severity (OR, 1.34; 95% CI, 1.15-1.58) and 30-day mortality (OR, 1.59; 95% CI, 1.25-2.02) among Black patients compared with White patients, after adjustment for baseline covariates (Table 3). The propensity scores for race were balanced and overlapping (eFigure 4 in Supplement 1). After balancing racial groups with respect to baseline covariates (eFigure 5 in Supplement 1), the OR for COVID-19 severity was 1.21 (95% CI, 1.11-1.33), and the relative risk of mortality was 1.14 (95% CI, 0.95-1.37) for Black patients compared with White patients (Table 3).

**Table 3.** Unweighted and Weighted Analyses of Association of Racial Disparities With COVID-19 Severity (Primary Outcome) and 30-Day All-Cause Mortality (Secondary Outcome)

Analysis	COVID-19 severity, OR (95% CI)^a^	30-d mortality	
		OR (95% CI)^b^	RR (95% CI)^c^
Unweighted			
Minimally adjusted^d^	1.50 (1.29-1.73)	1.71 (1.39-2.12)	1.52 (1.29-1.79)
Fully adjusted^e^	1.34 (1.15-1.58)	1.59 (1.25-2.02)	1.41 (1.19-1.65)
Inverse probability of treatment weighted^f^	1.21 (1.11-1.33)	1.16 (0.94-1.44)	1.14 (0.95-1.37)

Abbreviations: OR, odds ratio; RR, relative risk.

^a^ ORs comparing COVID-19 severity between non-Hispanic Black vs non-Hispanic White patients were estimated from ordinal logistic regression models; ORs greater than 1 indicate higher COVID-19 severity.

^b^ ORs comparing 30-day mortality between non-Hispanic Black vs non-Hispanic White patients were estimated from logistic regression models; ORs greater than 1 indicate higher odds of 30-day all-cause mortality.

^c^ RRs comparing 30-day mortality between non-Hispanic Black vs non-Hispanic White patients were estimated from modified Poisson regression models; RRs greater than 1 indicate higher risk of 30-day all-cause mortality.

^d^ Adjusted for age (linear and quadratic terms) and sex.

^e^ Adjusted for age (linear and quadratic terms), sex, region of patient residence, smoking status, obesity, cardiovascular and pulmonary comorbidities, kidney disease, diabetes, type of malignant neoplasm, Eastern Cooperative Oncology Group performance status, cancer status, timing and modality of anticancer therapy, and month of COVID-19 diagnosis.

^f^ Weighted by the reciprocal of the probability of receiving the treatment (ie, race) that was actually received, which was estimated from a propensity score model for race that included age, sex, region of patient residence, smoking status, obesity, cardiovascular and pulmonary comorbidities, kidney disease, diabetes, type of malignant neoplasm, Eastern Cooperative Oncology Group performance status, cancer status, timing and modality of anticancer therapy, and month of COVID-19 diagnosis. Inverse probability of treatment weighted analysis was conducted during manuscript revision.

Significant interactions were observed for obesity and race and for cancer status and race (Figure). In particular, obesity was associated with higher COVID-19 severity (OR, 1.38; 95% CI, 1.07-1.78) and 30-day mortality (OR, 1.54; 95% CI, 1.05-2.24) among Black patients, but obesity was not associated with these outcomes among White patients with cancer (COVID-19 severity, OR, 0.99; 95% CI, 0.82-1.18; 30-day mortality, OR, 0.92; 95% CI, 0.68-1.25). Actively progressing cancer was also significantly associated with increased COVID-19 severity among Black patients (OR, 4.55; 95% CI, 3.02-6.87) compared with White patients (OR, 2.76; 95% CI, 2.04-3.73). No other significant interactions were observed.

**Figure.**
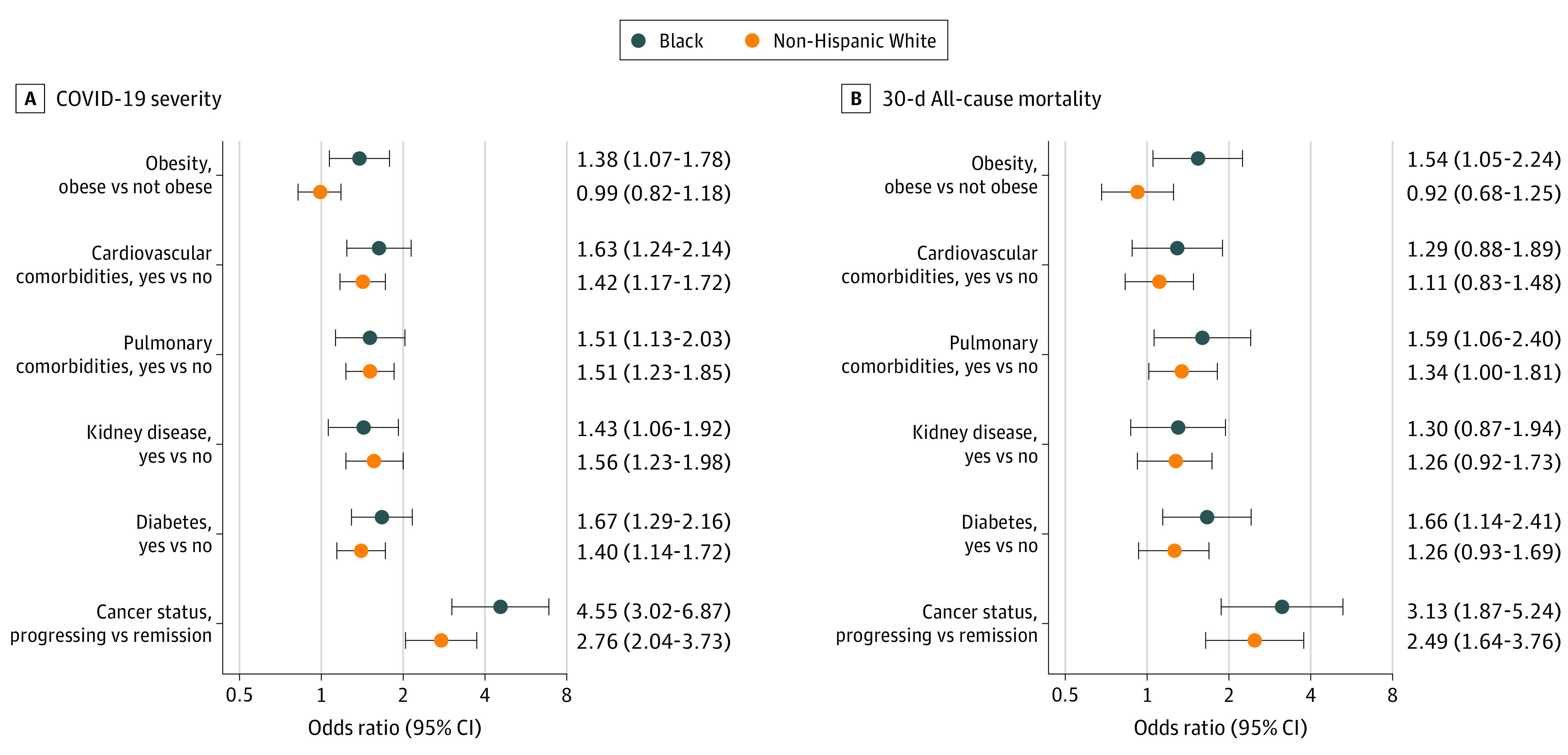
Adjusted Associations of Key Risk Factors With COVID-19 Severity and 30-Day All-Cause Mortality Stratified by Race and Ethnicity Data are shown for 3506 patients. Odds ratios (ORs) greater than 1 indicate higher COVID-19 severity or higher odds of 30-day all-cause mortality. ORs were adjusted for age (linear and quadratic terms), sex, region of patient residence, smoking status, obesity, cardiovascular and pulmonary comorbidities, kidney disease, diabetes, type of malignant neoplasm, Eastern Cooperative Oncology Group performance status, cancer status, timing and modality of anticancer therapy, and month of COVID-19 diagnosis. The contrast for cancer status is active and progressing cancer status vs remission or no evidence of disease; there was no evidence of effect modification for other categories (ie, active and responding, active and stable, unknown).

The e-value for the unweighted mortality OR was 1.83, and the e-value for the 95% CI was 1.48. Thus, an unobserved factor would need to be associated with both race and mortality with a risk ratio of at least 1.83 to fully attenuate the observed association; the risk ratio would need to be at least 1.48 for the null-hypothesized value (1.0) to be included in the 95% CI. Such an association is larger than most documented associations in the CCC19 cohort.^23^ The 95% CI from the weighted model for mortality included the null value.

## Discussion

The COVID-19 pandemic has highlighted and exacerbated longstanding health and social inequities in the US. These are likely the same inequities that fuel the disparities seen across the cancer control continuum and that lead to higher mortality rates among Black patients.^32^ Given the paucity of data on one of the most vulnerable racial and ethnic groups,^15,33^ in this cohort study, we examined a range of clinically meaningful characteristics and outcomes in Black patients with cancer and COVID-19 in the US. We used a novel ordinal outcome of COVID-19 severity to capture the full spectrum of COVID-19 complications that may vary across different racial and ethnic groups and also explored the interactions between comorbidities and cancer to understand their synergistic effect on COVID-19 outcomes in different racial groups.^34^

Compared with White patients, Black patients had worse COVID-19 presentations and experienced significantly higher COVID-19 severity; this difference was consistent across different analysis methods. These findings are complementary to a recent EHR report that showed African American patients with cancer and COVID-19 had higher rates of hospitalization and death.^12^ Our study validates and adds to their findings by examining a large cohort of Black patients with more granular data on the cancer status, severity of COVID-19 at presentation, course of illness, including systemic complications, and outcomes over longitudinal follow-up. Structural racism refers to the ways in which societies reinforce systems of health care, law enforcement, education, employment, benefits, media, and housing that perpetuate discriminatory distribution of resources and attitudes.^35,36^ The COVID-19 pandemic highlighted the health burden on racial and ethnic minority groups and the complexity of structural racism, which has led to disproportionately worse clinical outcomes among Black patients.^37,38^ Cancer health disparities have been well described elsewhere^6,18^; for example, Black women have a disproportionately higher breast cancer–specific mortality compared with White women, and Black men have a higher incidence and mortality from prostate cancer compared with White men. Factors such as access to care and SDOH that contribute to racial disparities in patients with cancer are complex and potentially extend to disparities in COVID-19 outcomes as well.

How should these findings be interpreted? The inequity in access to quality health care that can lead to worse baseline clinical factors and ultimately severe complications among racial and ethnic minority groups is well known.^6^ Our findings are similar to the patterns of racial disparities observed among patients with cancer, which points to an overlap in the root causes of racial inequities between cancer and COVID-19. In addition, racial and ethnic minority groups are more likely to live in conditions that pose a challenge to social distancing and also are more likely to be essential workers; therefore, sheltering in place may not be a possibility for many of them, thus putting these populations at an increased risk for exposure to SARS-CoV-2.^6,39^ These and additional sociodemographic pressures such as underinsurance may lead to delays in seeking medical care, which would likely be associated with more severe COVID-19 upon presentation.

Unfortunately, if these same racial inequities in access to medical care hold for cancer screening, in the near future, we are likely to see worsening disparities in rates of advanced stage cancers at diagnosis.^6^ The COVID-19 pandemic has been especially challenging for the treatment of patients with cancer.^40^ In our study, the racial disparities in COVID-19 severity were sustained for patients receiving active cancer therapy (Table 3). In addition, there are a host of drug interactions between chemotherapeutic agents and COVID-19 therapies, leading to alterations in standard treatments. For patients receiving radiation, the pandemic has caused delays between radiotherapy sessions, possibly leading to a reduction in therapeutic efficacy.^41^ Finally, current and other studies^23,42,43^ have demonstrated that Black patients with COVID-19 are less likely to receive novel anti–COVID-19 therapies (eg, remdesivir) compared with their White counterparts. Our findings that Black patients with cancer and COVID-19 were less likely to receive remdesivir, despite presenting with worse disease, may reflect a persistent gap in health care access and equity for Black patients. Unequal access and mistrust of the medical profession among vulnerable populations has resulted in lower rates of vaccine trial enrollment^44^ and COVID-19 vaccinations received in the Black community,^45^ all of which will further exacerbate racial disparities unless urgent remedial actions are undertaken.

### Strengths and Limitations

The current study has several strengths, including being, to our knowledge, the largest cohort to date of Black patients with cancer and COVID-19. Furthermore, we present data representative of Black patients across diverse age groups, cancer types, cancer status, geographical census regions, and academic and community centers, with longer-term follow-up beyond 30 days. Despite these strengths, there remain limitations associated with incomplete documentation of race and ethnicity, a commonly encountered problem in EHRs, and inherent lack of granularity in the Centers for Disease Control and Prevention classification schema chosen for the registry. We also did not examine the association of COVID-19 with cancer among Hispanic Black patients. Gender was collected in the survey, including a third nonbinary option. For the purposes of this analysis, we mapped gender to sex: woman to female, man to male, and nonbinary to unknown or missing. We acknowledge that this may lead to a mismatch between biological sex and gender identity, which is an inherent limitation of both the survey and underlying EHR data that is available to the respondents. Although a number of CCC19 participating sites are also National Cancer Institute Community Oncology Research Program sites, there is potentially incomplete capture of subpopulations of patients (eg, rural populations) who may not seek care at the mostly urban health care centers that compose the CCC19. In addition, CCC19 does not collect detailed data on SDOH, primarily because they are often not recorded within EHRs.^46^ Because insurance (a surrogate of health care access) is a contributor of disparity, it was considered as a nonallowable covariate and, thus, was not included in the analysis. A sensitivity analysis including insurance showed a marginal decrease in the OR for COVID-19 severity outcome and a negligible change in OR or relative risk for mortality (eTable 6 in Supplement 1). Thus, much if not all the disparity in COVID-19 severity between Black and White patients with cancer could be explained by measurable and sometimes modifiable factors.

## Conclusions

Our study found that Black patients with cancer and COVID-19 have similar cancer status but worse preexisting comorbidities, severity of COVID-19 at presentation, and outcomes compared with White patients with cancer and COVID-19. Understanding and addressing the cumulative and synergistic association of racial inequities (eg, preexisting comorbidities, SDOH, and inadequate access to quality health care and cutting-edge research) on clinical outcomes is pivotal. This is a call for action to eradicate root causes of racial inequities, within the causal framework of structural racism, to reduce the disproportionate burden of diseases, such as COVID-19 and cancer, among Black patients and, possibly, other minority racial and ethnic groups.

## Notes

Supplement 1. **eAppendix 1.** CCC19 Data Collection and Quality Assurance **eAppendix 2.** Alphabetical List of Participants by Institution that Contributed at Least One Record to the Analysis **eAppendix 3.** Statistical Analysis Plan **eAppendix 4.** Statistical Methods
eReferences
**eFigure 1.** Flow Diagram **eFigure 2.** COVID-19 Severity by Age **eFigure 3.** Differences in Outcome Log Odds Between Univariable Logistic Regression Models for All Possible Cutpoints of the Ordinal COVID-19 Severity Outcome, Relative to the ≥1 Versus 0 Comparison **eFigure 4.** Distribution and Summary Statistics for Propensity Scores **eFigure 5.** Absolute Standardized Mean Differences for Demographic and Clinical Characteristics at COVID-19 Diagnosis Between Non-Hispanic Black and Non-Hispanic White Patients **eTable 1.** Metrics for Data Quality **eTable 2.** Patients on Multimodal Anticancer Therapy **eTable 3.** Type of Malignancy **eTable 4.** Laboratory Measurements Among Hospitalized Patients **eTable 5.** Rates of Cardiovascular, Pulmonary, and Gastrointestinal Complications **eTable 6.** Inverse Probability Treatment Weighting (IPTW) With Insurance Added

Supplement 2.
The COVID-19 and Cancer Consortium (CCC19) Collaborators
