# Error in Supplement 2

**DOI:** 10.1001/jamanetworkopen.2022.17952

**Published:** 2022-06-01

In the Original Investigation titled “Racial Disparities in COVID-19 Outcomes Among Black and White Patients With Cancer,”^1^ published March 28, 2022, there was an incorrect name in Supplement 2, the nonauthor collaborator list. Pier Vitale should have appeared as Pier Vitale Nuzzo. This article has been corrected.^1^
