# Supplementary material for: Racial Disparities in COVID-19 Outcomes Among Black and White Patients With Cancer
Source: JAMA Netw Open. 2022 Mar 28;5(3):e224304. doi: 10.1001/jamanetworkopen.2022.4304 (PMC8961318; doi:10.1001/jamanetworkopen.2022.4304)
Supplement: Supplement 1. — eAppendix 1. CCC19 Data Collection and Quality Assurance eAppendix 2. Alphabetical List of Participants by Institution that Contributed at Least One Record to the Analysis eAppendix 3. Statistical Analysis Plan eAppendix 4. Statistical Methods eReferences eFigure 1. Flow Diagram eFigure 2. COVID-19 Severity by Age eFigure 3. Differences in Outcome Log Odds Between Univariable Logistic Regression Models for All Possible Cutpoints of the Ordinal COVID-19 Severity Outcome, Relative to the ≥1 Versus 0 Comparison eFigure 4. Distribution and Summary Statistics for Propensity Scores eFigure 5. Absolute Standardized Mean Differences for Demographic and Clinical Characteristics at COVID-19 Diagnosis Between Non-Hispanic Black and Non-Hispanic White Patients eTable 1. Metrics for Data Quality eTable 2. Patients on Multimodal Anticancer Therapy eTable 3. Type of Malignancy eTable 4. Laboratory Measurements Among Hospitalized Patients eTable 5. Rates of Cardiovascular, Pulmonary, and Gastrointestinal Complications eTable 6. Inverse Probability Treatment Weighting (IPTW) With Insurance Added [file jamanetwopen-e224304-s001.pdf]

Supplemental Online Content

Fu J, Reid SA, French B, et al; COVID-19 and Cancer Consortium (CCC19). Racial disparities in COVID-19 outcomes among Black and White patients with cancer. *JAMA Netw Open*. 2022;5(3):e224304. doi:10.1001/jamanetworkopen.2022.4304

eAppendix 1. CCC19 Data Collection and Quality Assurance
eAppendix 2. Alphabetical List of Participants by Institution that Contributed at Least One Record to the Analysis
eAppendix 3. Statistical Analysis Plan
eAppendix 4. Statistical Methods
eReferences
eFigure 1. Flow Diagram
eFigure 2. COVID-19 Severity by Age
eFigure 3. Differences in Outcome Log Odds Between Univariable Logistic Regression Models for All Possible Cutpoints of the Ordinal COVID-19 Severity Outcome, Relative to the ≥1 Versus 0 Comparison
eFigure 4. Distribution and Summary Statistics for Propensity Scores
eFigure 5. Absolute Standardized Mean Differences for Demographic and Clinical Characteristics at COVID-19 Diagnosis Between Non-Hispanic Black and Non-Hispanic White Patients
eTable 1. Metrics for Data Quality
eTable 2. Patients on Multimodal Anticancer Therapy
eTable 3. Type of Malignancy
eTable 4. Laboratory Measurements Among Hospitalized Patients
eTable 5. Rates of Cardiovascular, Pulmonary, and Gastrointestinal Complications
eTable 6. Inverse Probability Treatment Weighting (IPTW) With Insurance Added

This supplemental material has been provided by the authors to give readers additional information about their work.

© 2022 Fu J et al. *JAMA Network Open.*

eAppendix 1. CCC19 Data Collection and Quality Assurance

All information is retrieved from electronic health records and there is no direct contact with patients. With missing data, attempts to inquire further with active providers are pursued for completion of record. Information is abstracted from the electronic health records manually by designees of academic and community institutions. Each case report is assigned a “quality score,” which is a numeric metric to define case reports as analytic (0-4) or non-analytic (>4) quality based on data problems classified as minor, moderate, and major. The quality score is elaborated in our prior publication and summarized in eTable 1.[1] Only records meeting a sufficient quality score (0-4 points, i.e., no major problems and at most one moderate problem) are included (eFigure 1).

eAppendix 2. Alphabetical List of Participants by Institution that Contributed at Least One Record to the Analysis

Bolded = site PI/co-PIs; site co-investigators are listed alphabetically by last name.

Balazs Halmos, MD; Amit Verma, MBBS; Benjamin A. Gartrell, MD; Sanjay Goel, MBBS; Nitin Ohri, MD; R. Alejandro Sica, MD; Astha Thakkar, MD (Albert Einstein College of Medicine, Montefiore Medical Center, Bronx, NY, USA)

Keith Stockerl-Goldstein, MD; Omar Butt, MD, PhD; Jian L. Campian, MD, PhD; Mark A. Fiala, MSW; Ryan Monahan, MBA; Alice Y. Zhou, MD, PhD (Alvin J. Siteman Cancer Center at Washington University School of Medicine and Barnes-Jewish Hospital, St. Louis, MO, USA)

Michael A. Thompson, MD, PhD, FASCO; Pamela Bohachek, RN; Daniel Mundt, MD; Mitrianna Streckfuss, MPH; Eyob Tadesse, MD (Aurora Cancer Care, Advocate Aurora Health, Milwaukee, WI, USA)

Philip E. Lammers, MD, MSCI (Baptist Cancer Center, Memphis, TN, USA)

Sanjay G. Revankar, MD, FIDSA (The Barbara Ann Karmanos Cancer Institute at Wayne State University School of Medicine, Detroit, MI, USA)

Jaymin M. Patel, MD; Andrew J. Piper-Vallillo, MD; Poorva Bindal, MBBS (Beth Israel Deaconess Medical Center, Boston, MA, USA)

Orestis A. Panagiotou, MD, PhD; Pamela C. Egan, MD; Dimitrios Farmakiotis, MD, FACP, FIDSA; Hina Khan, MD; Adam J. Olszewski, MD (Brown University and Lifespan Cancer Institute, Providence, RI, USA)

Arturo Loaiza-Bonilla, MD, MSEd, FACP (Cancer Treatment Centers of America, AZ/GA/IL/OK/PA, USA)

Salvatore A. Del Prete, MD; Anne H. Angevine, MD; Michael H. Bar, MD, FACP; Anthony P. Gulati, MD; K. M. Steve Lo, MD; Jamie Stratton, MD; Paul L. Weinstein, MD (Carl & Dorothy Bennett Cancer Center at Stamford Hospital, Stamford, CT, USA)

Paolo Caimi, MD; Jill S. Barnholtz-Sloan, PhD; Jorge A. Garcia, MD, FACP; John M.

© 2022 Fu J et al. *JAMA Network Open.*

Nakayama, MD (Case Comprehensive Cancer Center at Case Western Reserve University/University Hospitals, Cleveland, OH, USA)

Shilpa Gupta, MD; Nathan A. Pennell, MD, PhD, FASCO; Manmeet S. Ahluwalia, MD, FACP; Scott J. Dawsey, MD; Christopher A. Lemmon, MD; Amanda Nizam, MD (Cleveland Clinic, Cleveland, OH, USA)

Claire Hoppenot, MD; Ang Li, MD, MS (Dan L Duncan Comprehensive Cancer Center at Baylor College of Medicine, Houston, TX, USA)

Toni K. Choueiri, MD; Ziad Bakouny, MD, MSc; Gabrielle Bouchard, BS; Fiona J. Busser, BA; Jean M. Connors, MD; Catherine R. Curran, BA; George D. Demetri, MD, FASCO; Antonio Giordano, MD, PhD; Kaitlin Kelleher, BA; Anju Nohria, MD; Andrew Schmidt, MD; Grace Shaw, BA; Eli Van Allen, MD; Pier Vitale Nuzzo, MD, PhD; Wenxin (Vincent) Xu, MD; Rebecca L. Zon, MD (Dana-Farber Cancer Institute, Boston, MA, USA)

Tian Zhang, MD, MHS; Susan Halabi, PhD, FASCO (Duke Cancer Institute at Duke University Medical Center, Durham, NC, USA)

John C. Leighton Jr, MD, FACP (Einstein Healthcare Network, Philadelphia, PA, USA)

Gary H. Lyman, MD, MPH, FASCO, FRCP; Jerome J. Graber MD, MPH; Petros Grivas, MD, PhD; Ali Raza Khaki, MD; Elizabeth T. Loggers, MD, PhD; Ryan C. Lynch, MD; Elizabeth S. Nakasone, MD, PhD; Michael T. Schweizer, MD; Lisa Tachiki, MD; Shaveta Vinayak, MD, MS; Michael J. Wagner, MD; Albert Yeh, MD (Fred Hutchinson Cancer Research Center/University of Washington/Seattle Cancer Care Alliance, Seattle, WA, USA)

Sharad Goyal, MD; Minh-Phuong Huynh-Le, MD, MAS (George Washington University, Washington, DC, USA)

Lori J. Rosenstein, MD (Gundersen Health System, WI, USA)

Peter Paul Yu, MD, FACP, FASCO; Jessica M. Clement, MD; Ahmad Daher, MD; Mark Dailey, MD; Rawad Elias, MD; Asha Jayaraj, MD; Emily Hsu, MD; Alvaro G. Menendez, MD; Joerg Rathmann, MD; Oscar Serrano, MD (Hartford HealthCare Cancer Institute, Hartford, CT, USA)

Clara Hwang, MD; Shirish M. Gadgeel, MD, Sunny R K Singh, MD (Henry Ford Cancer Institute, Henry Ford Hospital, Detroit, MI, USA)

Jessica E. Hawley, MD; Dawn Hershman, MD, MS, FASCO; Melissa K. Accordino, MD, MS; Divaya Bhutani, MD; Gary K. Schwartz, MD (Herbert Irving Comprehensive Cancer Center at Columbia University, New York, NY, USA)

Daniel Y. Reuben, MD, MS; Sarah Mushtaq, MD (Hollings Cancer Center at the Medical University of South Carolina, Charleston, SC, USA)

Eric H. Bernicker, MD (Houston Methodist Cancer Center, Houston, TX, USA)

John Deeken, MD; Danielle Shafer, DO (Inova Schar Cancer Institute, Fairfax, VA, USA)

© 2022 Fu J et al. *JAMA Network Open.*

Mark A. Lewis, MD; Terence D. Rhodes, MD, PhD; David M. Gill, MD; Clarke A. Low; MD (Intermountain Health Care, Salt Lake City, UT, USA)

Sandeep H. Mashru, MD; Abdul-Hai Mansoor, MD (Kaiser Permanente Northwest, OR/WA, USA)

Howard A. Zaren, MD, FACS; Stephanie J. Smith, RN, MSN, OCN (Lewis Cancer & Research Pavilion @ St. Joseph's/Candler, Savannah, GA, USA)

Gayathri Nagaraj, MD; Mojtaba Akhtari, MD; Eric Lau, DO; Mark E. Reeves, MD, PhD (Loma Linda University Cancer Center, Loma Linda, CA, USA)

Stephanie Berg, DO; Destry Elms, MD (Loyola University Medical Center, Maywood, IL, USA)

Alicia K. Morgans, MD, MPH; Firas H. Wehbe, MD, PhD; Jessica Altman, MD; Michael Gurley, BA; Mary F. Mulcahy, MD (Lurie Cancer Center at Northwestern University, Chicago, IL, USA)

Eric B. Durbin, DrPH, MS (Markey Cancer Center at the University of Kentucky, Lexington, KY, USA)

Amit A. Kulkarni, MD; Heather H. Nelson, PhD, MPH; Surbhi Shah, MD (Masonic Cancer Center at the University of Minnesota, Minneapolis, MN, USA)

Rachel P. Rosovsky, MD, MPH; Kerry Reynolds, MD; Aditya Bardia, MD; Genevieve Boland, MD, PhD, FACS; Justin Gainor, MD; Leyre Zubiri, MD, PhD (Massachusetts General Hospital Cancer Center, Boston, MA, USA)

Thorvardur R. Halfdanarson, MD; Tanios Bekaii-Saab, MD; Aakash Desai, MD, MPH; Zhuoer Xie, MD, MS (Mayo Clinic, AZ/FL/MN, USA)

Ruben A. Mesa, MD, FACP; Mark Bonnen, MD; Daruka Mahadevan, MD, PhD; Amelie G. Ramirez, DrPH, MPH; Mary Salazar, ANP; Dimpy P. Shah, MD, PhD; Pankil K. Shah, MD, MSPH (Mays Cancer Center at UT Health San Antonio MD Anderson Cancer Center, San Antonio, TX, USA)

Bryan Faller, MD (Missouri Baptist Medical Center, St. Louis, MO, USA)

Rana R. McKay, MD; Archana Ajmera, MSN, ANP-BC, AOCNP; Angelo Cabal, BS; Justin A. Shaya, MD (Moores Comprehensive Cancer Center at the University of California, San Diego, La Jolla, CA, USA)

Lisa B. Weissmann, MD, Chinmay Jani, MD (Mount Auburn Hospital, Cambridge, MA, USA)

Jeanna Knoble, MD; (Mary) Grace Glace, RN; Cameron Rink, PhD, MBA; Karen Stauffer, RN; Rosemary Zacks, RN (Mount Carmel Health System, Columbus, OH, USA)

Daniel G. Stover, MD; Daniel Addison, MD; James L. Chen, MD; Margaret E. Gatti-Mays, MD; Sachin

R. Jhawar, MD; Vidhya Karivedu, MBBS; Maryam B. Lustberg, MD, MPH; Joshua D. Palmer, MD; Clement Pillainayagam, MD; Sarah Wall, MD; Nicole Williams, MD (The Ohio State University Comprehensive Cancer Center, Columbus, OH, USA)

Monika Joshi, MD, MRCP; Harry Menon, DO, MPH; Marc A. Rovito, MD, FACP (Penn State Health/Penn State Cancer Institute/St. Joseph Cancer Center, PA, USA)

Elizabeth A. Griffiths, MD; Amro Elshoury, MBBCh (Roswell Park Comprehensive Cancer Center, Buffalo, NY, USA)

Salma K. Jabbour, MD; Mansi R. Shah, MD (Rutgers Cancer Institute of New Jersey at Rutgers Biomedical and Health Sciences, New Brunswick, NJ, USA)

Babar Bashir, MD, MS; Christopher McNair, PhD; Sana Z. Mahmood, BA, BS; Vasil Mico, BS; Chaim Miller, BA; Andrea Verghese Rivera, MD (Sidney Kimmel Cancer Center at Thomas Jefferson University, Philadelphia, PA, USA)

Sumit A. Shah, MD, MPH; Elwyn C. Cabebe, MD; Michael J. Glover, MD; Alokkumar Jha, PhD; Lidia Schapira, MD, FASCO; Julie Tsu-Yu Wu, MD, PhD (Stanford Cancer Institute at Stanford University, Palo Alto, CA, USA)

Suki Subbiah, MD (Stanley S. Scott Cancer Center at LSU Health Sciences Center, New Orleans, LA, USA)

Daniel B. Flora, MD, PharmD; Goetz Kloecker, MD; Barbara B. Logan, MS; Chaitanya Mandapakala, MD (St. Elizabeth Healthcare, Edgewood, KY, USA)

Gilberto de Lima Lopes Jr., MD, MBA, FAMS, FASCO (Sylvester Comprehensive Cancer Center at the University of Miami Miller School of Medicine, Miami, FL, USA)

Natasha Edwin, MD; Melissa Smits, APC (ThedaCare Cancer Care, Appleton, WI, USA)

David D. Chism, MD; Susie Owenby, RN, CCRP (Thompson Cancer Survival Center, Knoxville, TN, USA)

Deborah B. Doroshow, MD, PhD; Matthew D. Galsky, MD; Michael Wotman, MD; Huili Zhu, MD (Tisch Cancer Institute at the Icahn School of Medicine at Mount Sinai, New York, NY, USA)

Julie C. Fu, MD; Alyson Fazio, APRN-BC (Tufts Medical Center Cancer Center, Boston and Stoneham, MA, USA)

Jonathan Riess, MD, MS, Kanishka G. Patel, MD (UC Davis Comprehensive Cancer Center at the University of California at Davis, CA, USA)

Vadim S. Koshkin, MD; Daniel H. Kwon, MD (UCSF Helen Diller Family Comprehensive Cancer Center at the University of California at San Francisco, CA, USA)

© 2022 Fu J et al. *JAMA Network Open.*

Samuel M. Rubinstein, MD; William A. Wood, MD, MPH; Jessica Yasmine Islam, PhD, MPH; Vaibhav Kumar, MD (UNC Lineberger Comprehensive Cancer Center, Chapel Hill, NC, USA)

Trisha M. Wise-Draper, MD, PhD; Syed Ahmad, MD; Punita Grover, MD; Shuchi Gulati, MD; Jordan Kharofa, MD; Michelle Marcum, MS; Cathleen Park, MD (University of Cincinnati Cancer Center, Cincinnati, OH, USA)

Daniel W. Bowles, MD; Christoper L. Geiger, MD (University of Colorado Cancer Center, Aurora, CO, USA)

Merry-Jennifer Markham, MD, FACP, FASCO; Rohit Bishnoi, MD; Chintan Shah, MD (University of Florida Health Cancer Center, Gainesville, FL, USA)
Jared D. Acoba, MD; Young Soo Rho, MD, CM (University of Hawai'i Cancer Center, Honolulu, HI, USA)

Lawrence E. Feldman, MD; Kent F. Hoskins, MD; Gerald Gantt Jr., MD; Mahir Khan, MD; Ryan H. Nguyen, DO; Mary Pasquinelli, APN, DNP; Candice Schwartz, MD; Neeta K. Venepalli, MD, MBA (University of Illinois Hospital & Health Sciences System, Chicago, IL, USA)

Praveen Vikas, MD (University of Iowa Holden Comprehensive Cancer Center, Iowa City, IA, USA)

Elizabeth Wulff-Burchfield, MD; Anup Kasi MD, MPH (The University of Kansas Cancer Center, Kansas City, KS, USA)

Christopher R. Friese, PhD, RN, AOCN, FAAN; Leslie A. Fecher, MD (University of Michigan Rogel Cancer Center, Ann Arbor, MI, USA)

Blanche H. Mavromatis, MD; Ragneel Bijjula, MD; Qamar U. Zaman, MD (UPMC Western Maryland, Cumberland, MD, USA)

Jeremy L. Warner, MD, MS, FAMIA, FASCO; Alex Cheng, PhD; Elizabeth J. Davis, MD; Kyle T. Enriquez, MSc BS; Erin A. Gillaspie, MD, MPH; Daniel Hausrath, MD; Douglas B. Johnson, MD, MSCI; Xuanyi Li, BA; Sonya A. Reid, MD, MPH; Brian I. Rini, MD, FACP, FASCO; David A. Slosky, MD; Carmen C. Solorzano, MD, FACS; Matthew D. Tucker, MD (Vanderbilt-Ingram Cancer Center at Vanderbilt University Medical Center, Nashville, TN, USA)

Matthew Puc, MD; Theresa M. Carducci, MSN, RN, CCRP; Karen J. Goldsmith, BSN, RN; Susan Van Loon, RN, CTR, CCRP (Virtua Health, Marlton, NJ, USA)

Umit Topaloglu, PhD, FAMIA; Saif I. Alimohamed, MD (Wake Forest Baptist Comprehensive Cancer Center, Winston-Salem, NC, USA)

Robert L. Rice, MD, PhD (WellSpan Health, York, PA, USA)

Wilhelmina D. Cabalona, MD; Christine Pilar, BS, CCRC, ACRP-PM (Wentworth-Douglass Hospital, Dover, NH, USA)

© 2022 Fu J et al. *JAMA Network Open.*

Prakash Peddi, MD; Lane R. Rosen, MD; Briana Barrow McCollough, BSc, CCRC (Willis-Knighton Cancer Center, Shreveport, LA, USA)

Mehmet A. Bilen, MD; Deepak Ravindranathan, MD, MS (Winship Cancer Institute of Emory University, Atlanta, GA, USA)

Navid Hafez, MD, MPH; Roy Herbst, MD, PhD; Patricia LoRusso, DO, PhD; Tyler Masters, MS; Catherine Stratton, BA (Yale Cancer Center at Yale University School of Medicine, New Haven, CT, USA)

© 2022 Fu J et al. *JAMA Network Open.*

eAppendix 3. Statistical Analysis Plan

Approved Project Title	Racial and Ethnic Inequities and Disparities in Clinical Characteristics and Outcomes of Patients with Cancer and COVID - 19
Approved Project PI	Julie Fu
Name of the investigator completing this survey	Dimpy Shah
Proposed milestone deadline for this manuscript	9/3/20
Name and emails of (at most) 2 additional project team members who would like to be part of the analysis team for the project	Sonya Reid; Oscar Serrano
1 (a) Manuscript Title	Racial and Ethnic Inequities and Disparities in Clinical Characteristics and Outcomes of Patients with Cancer and COVID-19
1 (b) Provide in the abstract an informative and balanced summary of what was done and what will be found	The novel SARS-CoV2 virus and its resulting illness, COVID-19, has led to a global pandemic resulting in over 12 million cases worldwide and over 3 million cases in the United States (US).1 Initial reports implicate age, sex, and comorbid conditions as critical factors in determining the outcome from this illness. Most studies assessing outcomes of patients with cancer and COVID-19 have been limited by small sample size. One of the early reports from Wuhan, China reviewed 28 COVID-19-infected cancer patients with more than half experiencing severe outcomes and death in 28% of patients.2 It is postulated that cancer-directed treatment may be associated with severe events. These observations underscored the severity of COVID-19-infected patients with cancer and led to recommendations on COVID-19 screening and avoidance or dose modification of immunosuppressive treatments in these patients.2 Albeit limited, data from the US has corroborated worse outcomes following COVID-19 in patients with cancer.3,4 Based on recent disease-tracking dashboards, COVID-19 has been reported to disproportionately affect Blacks at higher rates compared to Non-Hispanic Whites (NHW).5 Blacks also have higher rates of hospitalization and death after contracting COVID-19.6-8 In New York City, Blacks had a substantially higher mortality rate (92.3 deaths per

© 2022 Fu J et al. *JAMA Network Open.*

	100,000) compared to Whites (45.2) and Asians (34.5).9 Similarly, in Chicago, Blacks accounted for 50% of COVID-19 cases and nearly 60% of COVID-19 related deaths even though they only account for 30% of the overall population.10 Similar observations of racial and ethnic disparities in impact of COVID-19 have been made in Louisiana,11 New Jersey,12 Georgia,13 Michigan14 and Connecticut.15 Despite these early reports, there is a paucity of dataon outcomes of COVID-19 infection in patients with cancer, stratified by race and ethnicity. Factors contributing to these disparities are complex and likely constitue an interplay of socioeconomic status, pre-existing comorbid conditions, cancer status at the time of infection, and access to care. Prior to COVID-19 pandemic, it was well known that Black patients with cancer have the highest death rates compared to all other racial and ethnic groups of patients with cancer.16 Given the higher rates of COVID-19-related mortality reported in minorities in the general population, we hypothesize that Black patients with cancer would have significantly worse outcomes than NHW patients with cancer, after accounting for confounding variables.
State specific objectives, including any prespecified hypotheses	1. Racial/Ethnic inequalities and inequities in baseline characteristics and severity of presentation at the time of COVID-19 Diagnosis (Non-Hispanic Blacks (NHB) versus NHW patients).<br><br>a. To identify disparities in demographic, socioeconomic, clinical characteristics (including status of cancer and anti-cancer treatment), and ECOG performance status) at the time of COVID-19 diagnosis between minority (NHB) and NHW patients with cancer.<br><br>b. To describe disparities in initial severity of COVID-19 infection at the time of presentation between minority (NHB) and NHW patients with cancer, within the context of racial inequalities.<br><br>2. Racial/Ethnic Disparities in Clinical Complications and Outcomes

© 2022 Fu J et al. *JAMA Network Open.*

	a. To assess disparities in COVID-19 severity (ordinal outcome) and 30-day all-cause mortality rate between minority (NHB) and NHW patients with cancer, after adjusting for other prognostic covariates. Hypothesis: Minority patients with cancer will have higher 30-day all-cause mortality rate compared to NHW patients with cancer.<br><br>b. To assess disparities in incidence of clinical complications (see appendix I) between minority (NHB) and NHW patients with cancer. Hypothesis: Minority patients with cancer will have significantly higher rates of clinical complications compared to NHW patients with cancer.<br><br>c. To assess disparities in rates of hospitalization, ICU admission, and mechanical ventilation between minority (NHB) and NHW patients with cancer. Hypothesis: Minority patients with cancer will have significantly higher rates of hospitalization, mechanical ventilation, and ICU admission compared to NHW patients with cancer.
Setting	The COVID-19 and Cancer Consortium (CCC19) (NCT04354701) is the largest international cohort study examining the clinical characteristics, course of illness, and outcomes of COVID-19 in patients with cancer. The CCC19 database is uniquely positioned to answer these critical and urgent questions to inform patients, caregivers, researchers, and policy makers about the burden of this pandemic on minorities. Data on all patients available at the time of analysis
(a) Give the eligibility criteria, and the sources and methods of selection of participants. Describe methods of follow-up	CCC19 records of all NHB and NHW patients with cancer and lab-confirmed SARS-CoV-2 diagnosis in US, and race/ethnicity data present at the time of the analyses. Each aim will be completed for the following groups (NHB vs. NHW): Age > 18 years<br><br>Exclusion criteria: Quality score >4<br>Non-invasive cancers and premalignant conditions<br>Non-melanoma non-invasive skin cancers (exclude if no confirmation)

© 2022 Fu J et al. *JAMA Network Open.*

	Incomplete follow-up resulting in unknown COVID-19 severity
(b) For matched studies, give matching criteria and number of exposed and unexposed	Not applicable
Outcomes	Objective 1a:<br><br>baseline demographic, socioeconomic, clinical characteristics (including status of cancer and anti-cancer treatment), comorbidity (cardiac, renal, pulmonary, diabetes), ECOG performance status, and severity of presentation of COVID-19<br><br>1b.<br>overall baseline health status (including clinical laboratory markers) between NHW and NHB in hospitalized patients<br><br><br>2a:<br>· Primary outcome measure will be ordinal variable with following COVID-19 severity (0=uncomplicated, 1=hospitalization, 2=ICU admission, 3=mechanical ventilation, 4=death). No time restriction for recording ordinal outcome<br>· Secondary outcome will be 30-day all-cause mortality for multivariable modeling<br><br>2b.<br>Simple summary table stratified by race that gives n(%) for:<br>1. clinical systemic complications (see appendix I)<br>2. total hospitalization<br>3. total mechanical ventilation<br>4. total ICU admission<br>5. overall Death
Exposures	Race (Black vs NHW)
Potential confounders	Age, gender, smoking, obesity, comorbidities (cardio, pulmonary, renal, diabetes), ECOG, cancer type/ status anti-cancer therapy, calendar time, census region of patient’s residence
Diagnostic criteria (if applicable)	N/A

© 2022 Fu J et al. *JAMA Network Open.*

For each variable of interest, give sources of data and details of methods of assessment (measurement). Describe comparability of assessment methods if there is more than one group	CCC19 database
Explain how the study size was arrived at	
Explain how quantitative variables will be handled in the analyses. If applicable, describe which groupings will be chosen and why	Age will be treated as continuous variable. Spline plot to identify cutoff thresholds. The remainder of the variables, including lab values, under examination are categorical in nature.
(a) Describe all statistical methods, including those to be used to control for confounding	Objective 1 will assess differences in baseline demographic, socioeconomic, comorbidities, clinical characteristics (including status of cancer and anti-cancer treatment), ECOG performance status, and severity of presentation of COVID-19 between each of the racial group comparisons. After checking for the accuracy, integrity, and distribution of the data, all characteristics and outcomes will be presented using descriptive statistics. We will provide the median and interquartile range (IQR) for continuous variables. Counts and percentages will be used to describe the binary and categorical variables.<br><br>1b. Descriptive table restricted to hospitalized patients: laboratory measurements<br><br>2a. Primary outcome measure will be ordinal variable and secondary outcome will be 30-d all-cause mortality for multivariable modeling. All *a priori* variables (but not baseline severity) and significant interactions will be included in the final MV model.<br><br>We will use the e value to quantify sensitivity to unmeasured confounding.<br><br>We will perform analysis based on inverse probability of treatment weighted (IPTW) methods. First, we will estimate propensity scores from a logistic regression model for which the outcome is a binary indicator of non-

© 2022 Fu J et al. *JAMA Network Open.*

	Hispanic Black versus non-Hispanic White race and prespecified covariates. For each patient, a weight will be calculated equal to the reciprocal of the probability of "receiving the treatment" (that is, race) that was "actually received," which will be estimated from the propensity score model.<br>Next, we will use graphics and summary statistics to evaluate the propensity score model. The empirical distributions of the propensity scores will be stratified by race will be plotted, to evaluate their overlap between groups. Mean propensity scores will be calculated stratified by race across quintiles of the propensity scores in the overall cohort, to evaluate balance in the propensity scores between groups. Unweighted and weighted absolute standardized mean differences for demographic and clinical characteristics at COVID-19 diagnosis between non-Hispanic Black and non-Hispanic White patients will be calculated, to evaluate whether the two groups were balanced on their observed characteristics; an absolute standardized mean difference <0.1 indicated balance.<br>Finally, we will estimate IPTW differences in COVID-19 severity between non-Hispanic Black and non-Hispanic White patients from an ordinal logistic regression model that included an offset for (log) follow-up time. Between-group IPTW differences in 30-day all-cause mortality will be estimated from both a logistic regression model (to estimate odds ratios) and a modified Poisson regression model (to estimate relative risks). All models will include race as the sole covariate, weighted by the reciprocal of the probability of "receiving the treatment" (that is, race) that was "actually received," and will use a robust (a.k.a. sandwich) variance estimator to account for the uncertainty due to estimation of the weights (and for the modified Poisson model, to account for misspecification of the variance structure). Results will be reported as odds ratios (or relative risks) with 95% confidence intervals.<br><br>Proportional odds assumption will be tested<br><br>2b.<br>Simple summary table stratified by race that gives n (%) for:<br>1. clinical systemic complications (see appendix I)

	2. total hospitalization<br>3. total mechanical ventilation<br>4. total ICU admission<br>5. overall Death
(b) Describe any methods that will be used to examine subgroups and interactions	We will also examine interaction between<br>1. race and all comorbidities (cardio, pulmonary, renal, diabetes), and<br>2. race and cancer status<br>3. race and obesity<br><br>to understand the synergistic impact of these factors on mortality.
(c) Explain how missing data will be addressed	Multiple imputation will be used to impute missing and unknown data for all variables included in the analysis, with some exceptions: unknown ECOG performance score and unknown cancer status will not be imputed and treated as a separate category in analyses; and laboratory values will not be imputed.<br><br>Imputation will be performed on the largest dataset possible (that is, after removing test cases and other manual exclusions, but before applying specific exclusion criteria). At least 10 imputed datasets will be used.
(d) If applicable, explain how loss to follow-up will be addressed	Not applicable
(e) Describe any sensitivity analyses	Not applicable
Complete?	Complete

Appendix I: <u>CCC19- Black vs White Clinical systemic complication</u>

Multisystem organ failure*	
Sepsis*	
Bleeding*	
DIC*	
Pulmonary complications*	Respiratory Failure<br>Pneumonitis<br>ARDS<br>Pulmonary embolism (PE)<br>Pleural effusion<br>Empyema

© 2022 Fu J et al. *JAMA Network Open.*

	Other, None, Unknown (?)
Cardiovascular complications*	Hypotension<br>Myocardial Infarction<br>Other cardiac ischemia<br>Atrial fibrillation<br>Ventricular fibrillation<br>Other cardiac arrhythmia<br>Cardiomyopathy<br>Congestive heart failure (CHF)<br>Pulmonary embolism (PE)<br>Deep venous thrombosis (DVT)<br>Superficial venous thrombosis (SVT)<br>Cerebrovascular accident (CVA; stroke)<br>Thrombosis, NOS<br>Other, None, Unknown (?)
Gastrointestinal complications*	Acute hepatic injury<br>Ascites<br>Bowel obstruction<br>Bowel perforation<br>Ileus<br>Peritonitis<br>Other, None, Unknown (?)
Other complications*	Acute Kidney injury (e.g. dialysis-the later my addition)<br>Seizures<br>Gangrene<br>Other, None, Unknown (?)
Supplemental O2 required (Y/N)*	
Coinfections (Y/N)*	
Blood transfusion (Y/N)*	

*Included in CCC19 data entry, no free text

SAP Finalization Date: 12/08/2020
SAP Revision Date: 07/15/2021

© 2022 Fu J et al. *JAMA Network Open.*

eAppendix 4. Statistical Methods

Regression models

Adjusted odds ratios (ORs) for COVID-19 severity were estimated from multivariable ordinal logistic regression models.[2] Because the ordinal outcome was assessed over patients' total follow-up period, the model included an offset for (log) follow-up time. Adjusted ORs and relative risks (RRs) for 30-day mortality were estimated from logistic and modified Poisson regression models, respectively.[3] In addition to models minimally adjusted for age and sex, we included all pre-specified covariates in fully adjusted models, given a sufficient number of events (and corresponding degrees of freedom) to enable full multivariable models. Coefficients and standard errors from models with different levels of adjustment, variance inflation factors, and clinical judgement were used to assess model stability. Exploratory analyses with smoothing splines were used to determine the association of age (as a continuous variable) with outcomes, which appeared non-linear (eFigure 2). Linear and quadratic terms for age (centered at 40 years) provided an adequate fit. All other covariates were categorical and were adjusted for using indicator variables for each category other than the reference category. These specifications reflected the assumed functional form for covariates. Note that these unweighted models quantified conditional differences in outcomes between non-Hispanic Black and non-Hispanic White patients, conditional on covariate values.

Upon revision, we performed analyses based on inverse probability of treatment weighted (IPTW) methods.[4] While some authors advocate for the use of methods based on causal inference to assess disparities,[5] others do not recommend these methods when the exposure of interest is intrinsic and not modifiable, which therefore does not allow a meaningful definition for counterfactual outcomes.[6] Because race as recorded in medical records and utilized in this analysis is a social and political construct, it is in theory a modifiable risk factor.[7]

First, we estimated propensity scores from a logistic regression model for which the outcome was a binary indicator of non-Hispanic Black versus non-Hispanic White race and the minimum sufficient adjustment set of covariates[5] including age, sex, region of patient residence, smoking status, obesity, cardiovascular and pulmonary comorbidities, renal disease, diabetes mellitus, type of malignancy, ECOG performance status, cancer status, timing and modality of anti-cancer therapy, and month of COVID-19 diagnosis, region of patient's residence, and calendar time, and without (primary) and with (sensitivity) insurance (with missing or unknown included as an "unknown" category). For each patient, a weight was calculated equal to the reciprocal of the probability of "receiving the treatment" (that is, race) that was "actually received," which was estimated from the propensity score model.

Next, we used graphics and summary statistics to evaluate the propensity score model.[8] The empirical distributions of the propensity scores stratified by race were plotted, to evaluate their overlap between groups. Mean propensity scores were calculated stratified by race across quintiles of the propensity scores in the overall cohort, to evaluate balance in the propensity scores between groups. Unweighted and weighted absolute standardized mean differences for demographic and clinical characteristics at COVID-19 diagnosis between non-Hispanic Black and non-Hispanic White patients were calculated, to evaluate whether the two groups were balanced on their observed characteristics; an absolute standardized mean difference <0.1 indicated balance.

© 2022 Fu J et al. *JAMA Network Open.*

Finally, we estimated IPTW differences in COVID-19 severity between non-Hispanic Black and non-Hispanic White patients from an ordinal logistic regression model that included an offset for (log) follow-up time. Between-group IPTW differences in 30-day all-cause mortality were estimated from both a logistic regression model (to estimate odds ratios) and a modified Poisson regression model (to estimate relative risks).[3] All models included race as the sole covariate, were weighted by the reciprocal of the probability of "receiving the treatment" (that is, race) that was "actually received," and used a robust (a.k.a. sandwich) variance estimator to account for the uncertainty due to estimation of the weights (and for the modified Poisson model, to account for misspecification of the variance structure). Results were reported as odds ratios (or relative risks) with 95% confidence intervals. Note that these weighted models quantified marginal differences in outcomes between non-Hispanic Black and non-Hispanic White patients.

Proportional odds assumption

We evaluated the proportional odds assumption by fitting a set of univariable logistic regression models for all possible cut points of the ordinal COVID-19 severity outcome, with:

Death from any cause:	4
Received mechanical ventilation:	3
Admitted to an intensive care unit:	2
Admitted to the hospital:	1
No complications:	0

That is, for each covariate, we fit a univariable logistic regression model with an offset for (log) follow-up time for each of the four binary outcomes of 4 versus <4, ≥3 versus <3, ≥2 versus <2, and ≥1 versus 0.[9] From each logistic regression model, we obtained the estimated logits (i.e., the log odds of the outcome) for all levels of the covariate. The estimated logits obtained from the 4 versus <4, ≥3 versus <3, and ≥2 versus <2 models were compared to those obtained from the ≥1 versus 0 model via subtraction, plotted, and visually inspected. If the proportional odds assumption was satisfied, then these logit differences would be similar (that is, "proportional") across all covariate levels. There did not appear to be systematic violations of the proportional odds assumption (eFigure 3), including for race; there was a suggestion that the assumption might not be satisfied for Eastern Cooperative Oncology Group (ECOG) performance status.

Missing data

Missing or unknown data for prognostic factors and other covariates could arise due to respondent non-response for optional survey questions or a response of unknown; an unknown category was provided for all survey questions. Therefore, we assumed that any missing or unknown data were, at worst, missing at random (i.e., missingness depends on observed data only); these missing or unknown data were imputed as described below. However, unknown ECOG performance status and cancer status could be due to the lack of reassessment after initiating new anti-cancer therapy, mixed findings on scans, and lack of surveillance, among other reasons. Therefore, unknown status could be related to unobserved data (that is, missing not at random), and not appropriate to impute. Instead, unknown ECOG performance status and unknown cancer status were included as "unknown" categories.

Multiple imputation using additive regression, bootstrapping, and predictive mean matching was used to impute missing and unknown data.[10] The imputation model included separate binary

variables for components of the ordinal COVID-19 severity outcome (hospital admission, intensive care unit admission, receipt of mechanical ventilation, all-cause mortality), race and ethnicity, other prognostic factors (age, sex, region of patient residence, smoking status, obesity, cardiovascular and pulmonary comorbidities, renal disease, diabetes mellitus, type of malignancy, Eastern Cooperative Oncology Group (ECOG) performance status, cancer status, timing and modality of anti-cancer therapy, and month of COVID-19 diagnosis), and anti-COVID-19 treatments (remdesivir, hydroxychloroquine, corticosteroids, and other). Because rates of missingness were <5% for all variables considered (Table 1), we generated 10 imputed datasets. Results were combined across these imputed datasets using Rubin's rules. Imputation was performed on the full dataset prior to applying exclusion criteria (n=4,965).

eReferences

1. The COVID-19 and Cancer Consortium. A systematic framework to rapidly obtain data on patients with cancer and COVID-19: CCC19 governance, protocol and quality assurance. Cancer Cell 2020; 38:761-6.
2. Walker SH, Duncan DB. Estimation of the probability of an event as a function of several independent variables. Biometrika 1967;54:167-79.
3. Zou G. A modified Poisson regression approach to prospective studies with binary data. Am J Epidemiol 2004;159: 702-6.
4. Rosenbaum PR, Rubin DB. The central role of the propensity score in observational studies for causal effects. Biometrika 1983;70: 41-55.
5. Duan N, Meng XL, Lin JY, Chen CN, Alegria M. Disparities in defining disparities: Statistical conceptual frameworks. Stat Med 2008;27: 3941-56.
6. Höfler M. Causal inference based on counterfactuals. BMC Med Res Methodol 2005;5: 28.
7. Kaufman J. Epidemiologic analysis of racial/ethnic disparities: some fundamental issues and a cautionary example. Soc. Sci. Med. 2008;66: 1659-1669.
8. Austin PC, Stuart EA. Moving towards best practice when using inverse probability of treatment weighting (IPTW) using the propensity score to estimate causal treatment effects in observational studies. Stat Med 2015;34: 3661-79.
9. Harrell FE. Regression Modeling Strategies: With Applications to Linear Models, Logistic Regression, and Survival Analysis. New York: Springer-Verlag, 2001.
10. White IR, Royston P, Wood AM. Multiple imputation using chained equations: Issues and guidance for practice. Stat Med 2011;30: 377-99.

© 2022 Fu J et al. *JAMA Network Open.*

eFigure 1. Flow Diagram

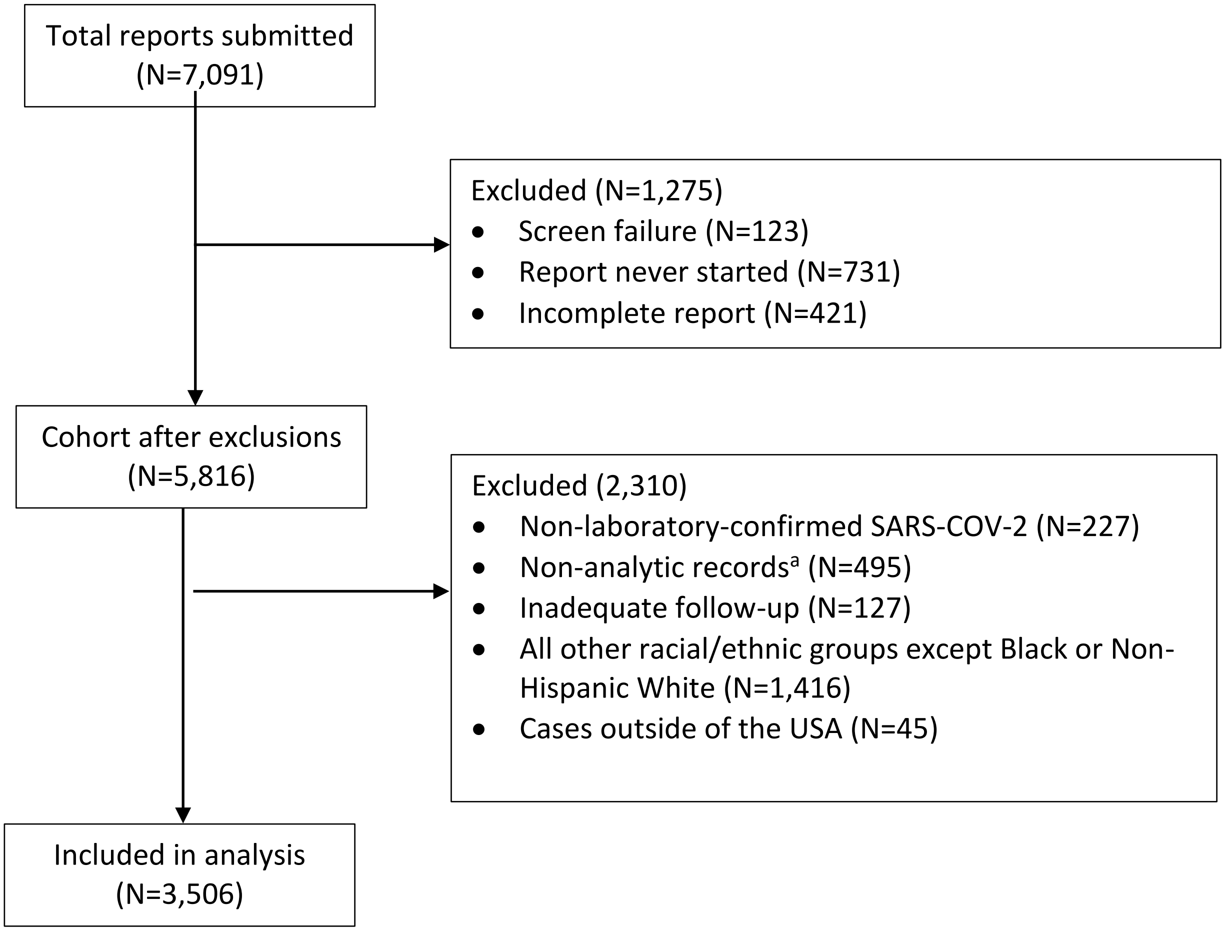

[a] Non-analytic records are case reports that did not meet data-quality metrics (eTable 1).

© 2022 Fu J et al. *JAMA Network Open.*

eFigure 2. COVID-19 Severity by Age[a]

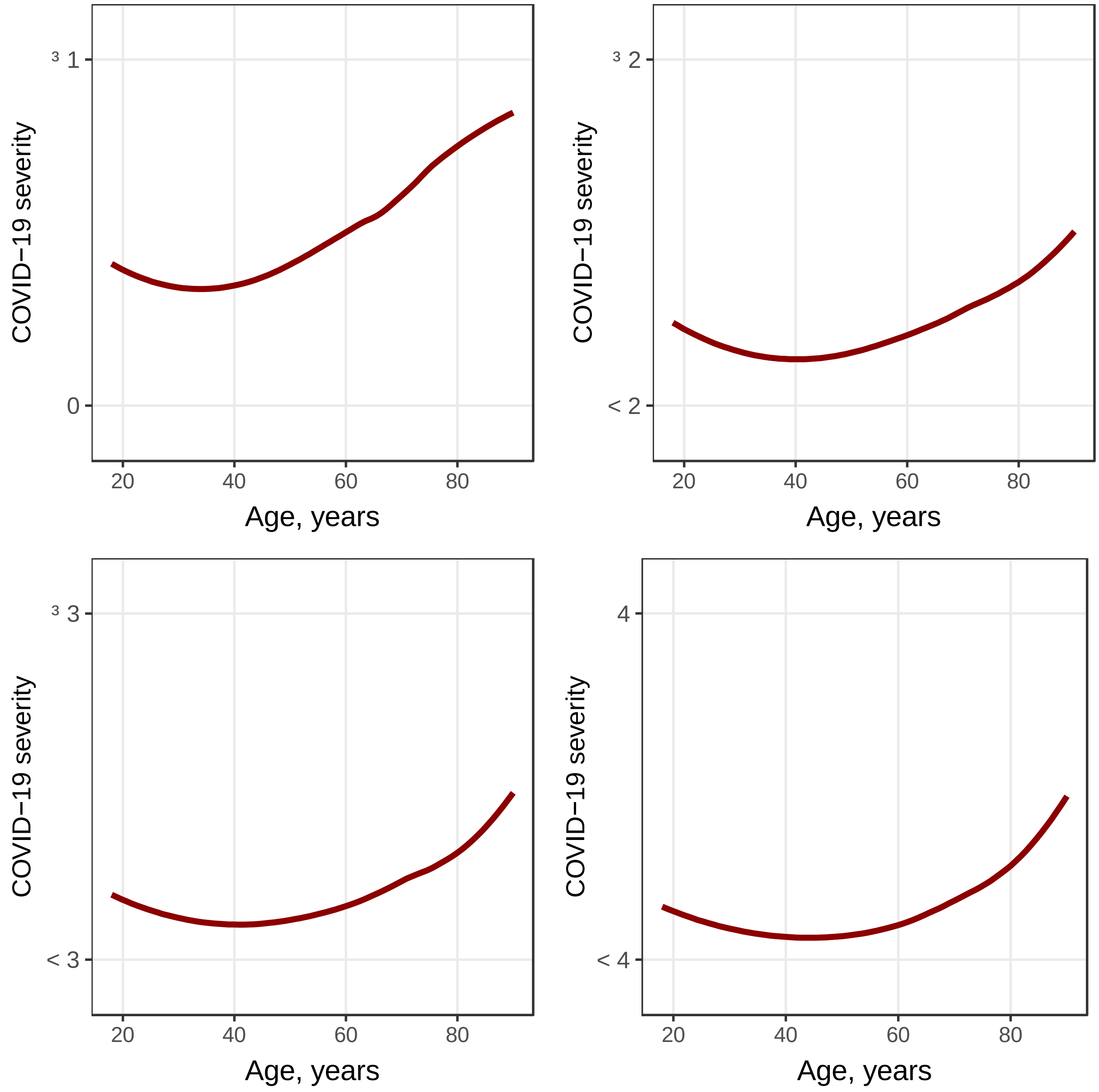

[a] Levels of ordinal COVID-19 severity are: 0, none of the following complications; 1, admitted to the hospital; 2, admitted to an intensive care unit; 3, received mechanical ventilation; 4, died from any cause. Points are jittered vertically to enhance legibility. Red lines and shaded regions represent LOESS smoothers and 95% confidence bands, respectively.

© 2022 Fu J et al. *JAMA Network Open.*

eFigure 3. Differences in Outcome Log Odds Between Univariable Logistic Regression Models for All Possible Cutpoints of the Ordinal COVID-19 Severity Outcome, Relative to the ≥1 Versus 0 Comparison[a]

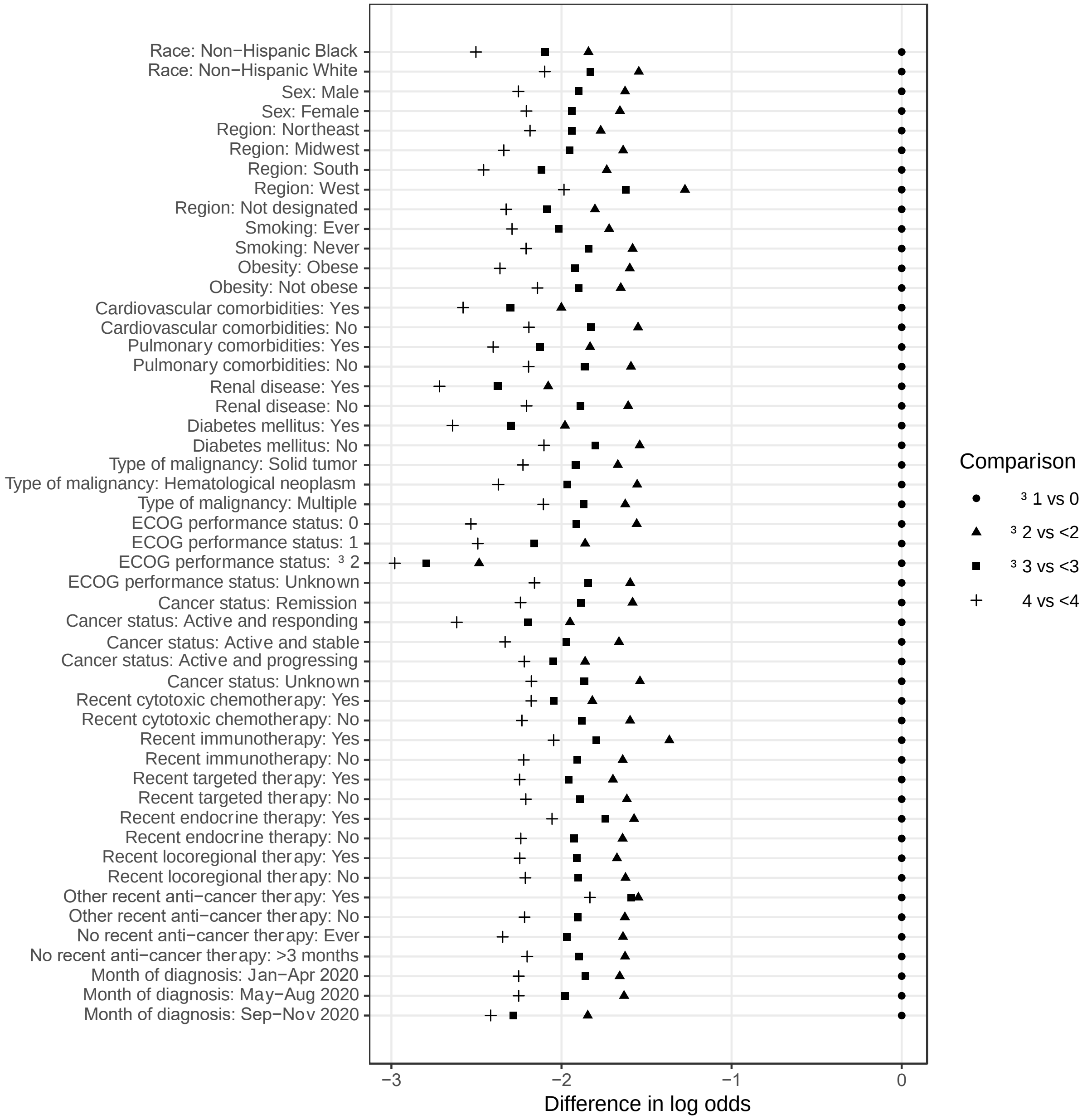

ECOG, Eastern Cooperative Oncology Group.

[a] Levels of ordinal COVID-19 severity are: 0, none of the following complications; 1, admitted to the hospital; 2, admitted to an intensive care unit; 3, received mechanical ventilation; 4, died from any cause.

© 2022 Fu J et al. *JAMA Network Open.*

eFigure 4. Distribution and Summary Statistics for Propensity Scores[a]

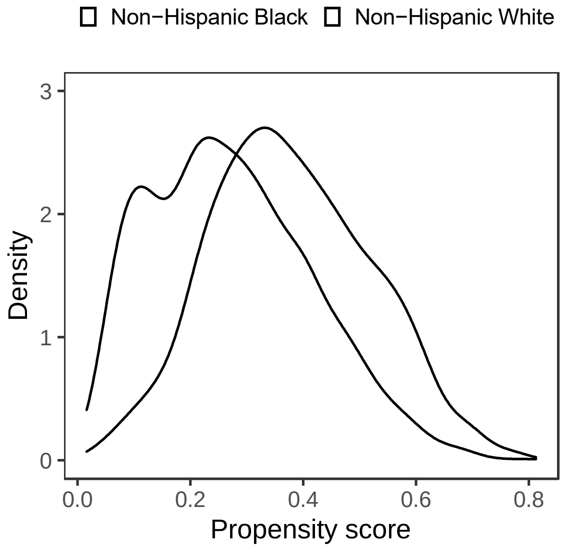

Percentile[a]	Mean propensity score Black	White
0–<20th	0.1139	0.1025
20–<40th	0.2181	0.2117
40–<60th	0.2969	0.2938
60–<80th	0.3841	0.3817
80–100th	0.5396	0.5177

[a] Percentiles (i.e., quintiles) of propensity scores in the total cohort.

[a] Propensity scores were estimated from a logistic regression model for race that included age, sex, region of patient residence, smoking status, obesity, cardiovascular and pulmonary comorbidities, renal disease, diabetes mellitus, type of malignancy, Eastern Cooperative Oncology Group performance status, cancer status, timing and modality of anti-cancer therapy, and month of COVID-19 diagnosis.

© 2022 Fu J et al. *JAMA Network Open.*

eFigure 5. Absolute Standardized Mean Differences for Demographic and Clinical Characteristics at COVID-19 Diagnosis Between Non-Hispanic Black and Non-Hispanic White Patients[a]

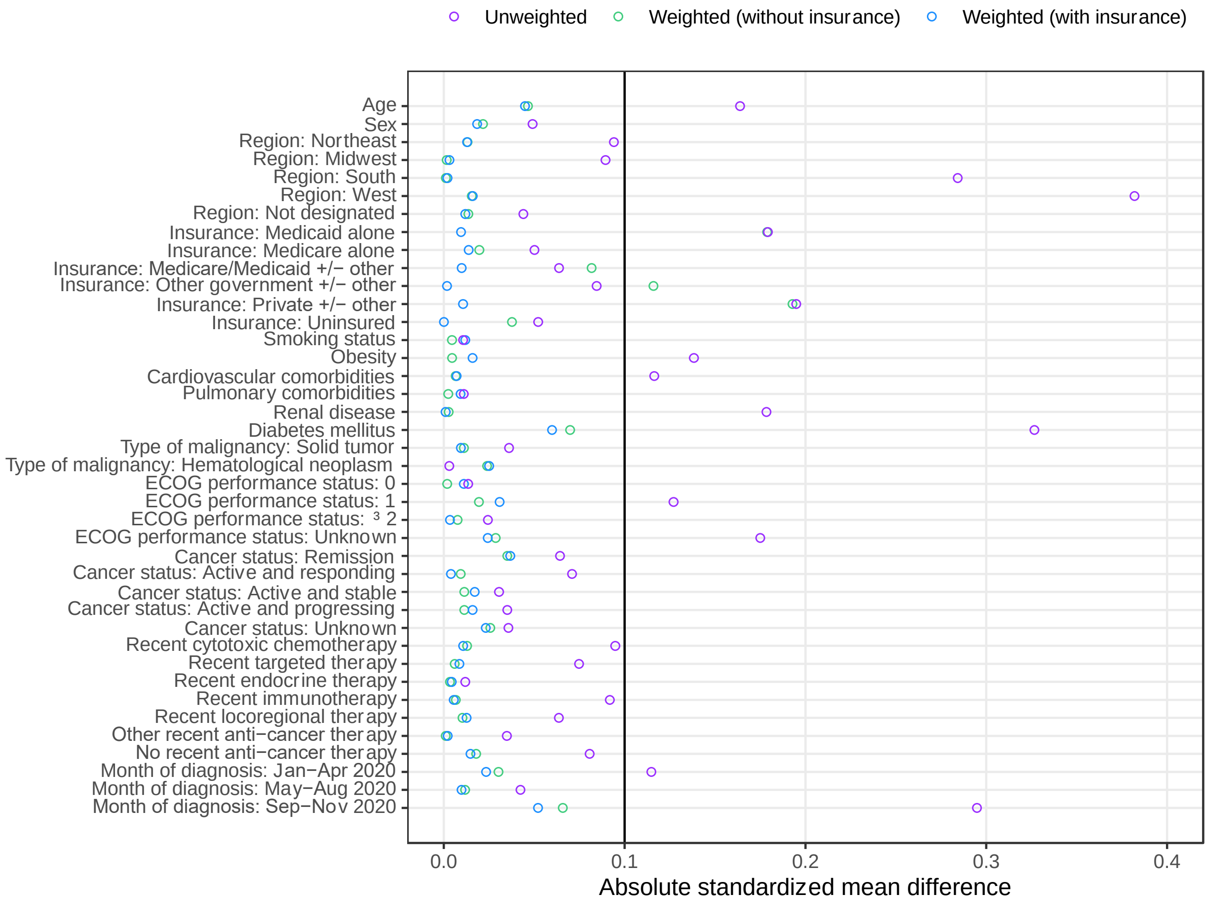

ECOG, Eastern Cooperative Oncology Group.

[a] Weighted absolute standardized mean differences were weighted by the reciprocal of the probability of “receiving the treatment” (that is, race) that was “actually received,” which was estimated from a propensity score model for race that included age, sex, region of patient residence, smoking status, obesity, cardiovascular and pulmonary comorbidities, renal disease, diabetes mellitus, type of malignancy, Eastern Cooperative Oncology Group performance status, cancer status, timing and modality of anti-cancer therapy, and month of COVID-19 diagnosis, and without (primary) and with (sensitivity) insurance.

© 2022 Fu J et al. *JAMA Network Open.*

eTable 1. Metrics for Data Quality

Quality score	Definition
0	No problems identified
1	1 minor problem
2	2 minor problems
3	3 minor problems or 1 moderate problem
4	4 minor problems or 1 moderate problem and 1 minor problem
5	5 minor problems or 1 moderate problem and 2 minor problems or 1 major problem
≥6	As above with additional problems

Minor problems were valued at 1 point, moderate problems at 3 points, and major problems at 5 points. Reports with a quality score of >4 were excluded from the analysis.

© 2022 Fu J et al. *JAMA Network Open.*

eTable 2. Patients on Multimodal Anticancer Therapy

	Targeted	Endocrine	Immunotherapy	Local	Other
Cyto	812	812	613	686	527
Targeted	-	741	590	713	465
Endocrine	-	-	502	591	368
Immunotherapy	-	-	-	452	188
Local	-	-	-	-	330

© 2022 Fu J et al. *JAMA Network Open.*

eTable 3. Type of Malignancy

	Total (N = 3506)	Non-Hispanic Black (N = 1068)	Non-Hispanic White (N = 2438)
Solid tumors, n (%)			
Breast	707 (20)	232 (22)	475 (19)
Prostate	593 (17)	211 (20)	382 (16)
Gastrointestinal	436 (12)	137 (13)	299 (12)
Other genitourinary	293 (8)	72 (7)	221 (9)
Thoracic	287 (8)	85 (8)	202 (8)
Gynecological	223 (6)	66 (6)	157 (6)
Endocrine	168 (5)	50 (5)	118 (5)
Skin cancer	153 (4)	6 (1)	147 (6)
Head and neck	103 (3)	28 (3)	75 (3)
Sarcoma	79 (2)	17 (2)	62 (3)
Nervous system	51 (1)	8 (1)	43 (2)
Not otherwise specified	38 (1)	17 (2)	21 (1)
Hematological neoplasms, n (%)			
Lymphoid neoplasms	428 (12)	106 (10)	322 (13)
Multiple myeloma	166 (5)	81 (8)	85 (3)
Myeloid neoplasm	152 (4)	43 (4)	109 (4)
Not otherwise specified	11 (<1)	3 (<1)	8 (<1)

Data presented as n (%). Categories are not mutually exclusive.

eTable 4. Laboratory Measurements Among Hospitalized Patients[a]

	Total (N = 3506)	Non-Hispanic Black (N = 1068)	Non-Hispanic White (N = 2438)
Absolute lymphocyte count, n (%)			
Low[b]	973 (48)	329 (47)	644 (48)
Normal	626 (31)	248 (36)	378 (28)
High[c]	56 (3)	12 (2)	44 (3)
Missing/unknown	371 (18)	107 (15)	264 (20)
Absolute neutrophil count, n (%)			
Low[c]	145 (7)	52 (7)	93 (7)
Normal	1207 (60)	449 (65)	758 (57)
High[c]	346 (17)	101 (15)	245 (18)
Missing/unknown	328 (16)	94 (14)	234 (18)
Platelet count, n (%)			
Low[c]	530 (26)	160 (23)	370 (28)
Normal	1177 (58)	438 (63)	739 (56)
High[c]	83 (4)	26 (4)	57 (4)
Missing/unknown	236 (12)	72 (10)	164 (12)
Creatinine, n (%)			
Normal	1016 (50)	292 (42)	724 (54)
Abnormal[c]	789 (39)	339 (49)	450 (34)
Missing/unknown	221 (11)	65 (9)	156 (12)
D-dimer, n (%)			
Normal	159 (8)	48 (7)	111 (8)
Abnormal[c]	954 (47)	364 (52)	590 (44)
Missing/unknown	913 (45)	284 (41)	629 (47)
Troponin, n (%)			
Normal	693 (34)	252 (36)	441 (33)
Abnormal[c]	474 (23)	151 (22)	323 (24)

© 2022 Fu J et al. *JAMA Network Open.*

Missing/unknown	859 (42)	293 (42)	566 (43)
Lactate dehydrogenase, n (%)			
Normal	244 (12)	74 (11)	170 (13)
Abnormal[c]	808 (40)	316 (45)	492 (37)
Missing/unknown	974 (48)	306 (44)	668 (50)
C-reactive protein, n (%)			
Normal	83 (4)	30 (4)	53 (4)
Abnormal[c]	1041 (51)	390 (56)	651 (49)
Missing/unknown	902 (45)	276 (40)	626 (47)

Data presented as n (%).

[a] Respondents were instructed to report the earliest measured laboratory measurements during COVID-19 course. Except for low absolute lymphocyte count (ALC), which was centrally defined as ALC < 1500/µL, ascertainment of upper and lower limits of normal was left to the discretion of respondents. Laboratory measurements were summarized among hospitalized patients only due to common clinical practice to avoid a laboratory blood draw for outpatients.

[b] Low absolute lymphocyte count is defined as less than 1500/uL.

[c] As defined by the reporting institution's normal laboratory value ranges.

© 2022 Fu J et al. *JAMA Network Open.*

eTable 5. Rates of Cardiovascular, Pulmonary, and Gastrointestinal Complications (N = 3506)

	Total		Non-Hispanic Black		Non-Hispanic White	
	N[a]	n (%)	N[a]	n[b] (%)	N[a]	n[b] (%)
Cardiovascular complications						
Hypotension	3373	401 (12)	1026	151 (15)	2347	250 (11)
Myocardial infarction	3365	47 (1)	1024	20 (2)	2341	27 (1)
Other cardiac ischemia	3365	31 (1)	1024	7 (1)	2341	24 (1)
Atrial fibrillation	3372	206 (6)	1025	50 (5)	2347	156 (7)
Ventricular fibrillation	3364	14 (<1)	1024	7 (1)	2340	7 (<1)
Other cardiac arrhythmia	3366	90 (3)	1026	34 (3)	2340	56 (2)
Cardiomyopathy	3365	24 (1)	1024	7 (1)	2341	17 (1)
Congestive heart failure	3365	113 (3)	1024	35 (3)	2341	78 (3)
Pulmonary embolism	3440	81 (2)	1048	30 (3)	2392	51 (2)
Deep venous thrombosis	3365	68 (2)	1024	25 (2)	2341	43 (2)
Superficial venous thrombosis	3365	11 (<1)	1024	<5 (<1)	2341	7 (<1)
Cerebrovascular accident	3365	40 (1)	1024	18 (2)	2341	22 (1)
Thrombosis, NOS	3395	24 (1)	1034	7 (1)	2361	17 (1)
Pulmonary complications						
Respiratory failure	3438	1002 (29)	1051	357 (34)	2387	645 (27)
Pneumonitis or pneumonia[c]	3420	440 (13)	1044	127 (12)	2376	313 (13)
ARDS	3425	275 (8)	1046	116 (11)	2379	159 (7)
Pulmonary embolism	3440	81 (2)	1048	30 (3)	2392	51 (2)
Pleural effusion	3422	135 (4)	1043	45 (4)	2379	90 (4)
Empyema	3418	8 (<1)	1043	<5 (<1)	2375	5 (<1)
Gastrointestinal complications						
Acute hepatic injury	3334	84 (3)	1015	36 (4)	2319	48 (2)
Ascites	3334	18 (1)	1016	8 (1)	2318	10 (<1)
Bowel obstruction	3333	15 (<1)	1015	5 (<1)	2318	10 (<1)
Bowel perforation	3332	6 (<1)	1015	0 (0)	2317	6 (<1)
Ileus	3332	12 (<1)	1015	6 (1)	2317	6 (<1)
Peritonitis	3332	6 (<1)	1015	<5 (<1)	2317	5 (<1)

ARDS, Acute respiratory distress syndrome; NOS, not otherwise specified.

[a] Number of patients with non-missing data.

[b] Groups with fewer than 5 patients were masked (i.e., <5) to minimize the risk of re-identification as per CCC19 policy.

[c] These are collected as separate complications but given the difficulty in radiographically distinguishing pneumonia from pneumonitis, they are combined here.

© 2022 Fu J et al. *JAMA Network Open.*

eTable 6. Inverse Probability Treatment Weighting (IPTW) With Insurance Added

COVID-19 severity		30-day mortality			
Odds Ratio	CI	Odds Ratio	CI	Relative Risk	CI
1.13	(1.02 - 1.24)	1.18	(0.95 - 1.47)	1.16	(0.96 - 1.39)

© 2022 Fu J et al. *JAMA Network Open.*
